# Supplementary material for: New sulphonamide pyrolidine carboxamide derivatives: Synthesis, molecular docking, antiplasmodial and antioxidant activities
Source: PLoS One. 2021 Feb 24;16(2):e0243305. doi: 10.1371/journal.pone.0243305 (PMC7904193; doi:10.1371/journal.pone.0243305)
Supplement: S1 File — (DOCX) [file pone.0243305.s001.docx]

**Synthesis and evaluation of new sulphonamide pyrolidine carboxamide as potential *Plasmodium falciparum* N-myristoyl transferase ligands and antiplasmodial agents**

Efeturi A. Onoabedje^a,c,*^, Akachukwu Ibezim^b,*^, Uchechukwu C. Okoro^a^, Sanjay Batra^c^

^a^Department of Pure & Industrial Chemistry, Faculty of Physical Sciences, University of Nigeria, Nsukka, Enugu State, Nigeria.

^b^Department of Pharmaceutical and Medicinal Chemistry, Faculty of Pharmaceutical Sciences, University of Nigeria, Nsukka, Enugu State, Nigeria.

^c^Division of Medicinal & Process Chemistry, Central Drug Research Institute, Lucknow, UP, India.

^*^Corresponding authors’ emails: [efeturi.onoabedje@unn.edu.ng](mailto:efeturi.onoabedje@unn.edu.ng) & [akachukwu.ibezim@unn.edu.ng](mailto:akachukwu.ibezim@unn.edu.ng)

**^1^H NMR, ^13^C NMR & Mass spectra**

**Material and methods**

General Information

Unless otherwise stated all reactions were performed in non-dry glassware under an air atmosphere and were monitored by analytical thin layer chromatography (TLC). TLC was performed on pre-coated silica gel plates. After elution, plate was visualized under UV illumination at 254 nm for UV active materials. The melting points were recorded on a hot stage apparatus and are uncorrected. IR spectra were recorded using a FTIR spectrophotometer. ^1^H NMR and ^13^C NMR spectra were recorded on 400 MHz NMR spectrometers with DMSO-*d*^6^ as solvent, using TMS as an internal standard (chemical shifts in ppm). Peak multiplicities of ^1^H-NMR signals were designated as s (singlet), brs (broad singlet), d (doublet), dd (doublet of doublet), t (triplet), q (quartet), p (pentet) m (multiplet) etc. Coupling constants (*J*) are in Hz. The ESI-MS were recorded on triple quadrupole Mass spectrometer. Column chromatography was performed using silica gel (100-200 mesh). Analytical grade solvents for the column chromatography were used as received. All the synthesised target compounds possesses purity of between 98 -100% in HPLC.


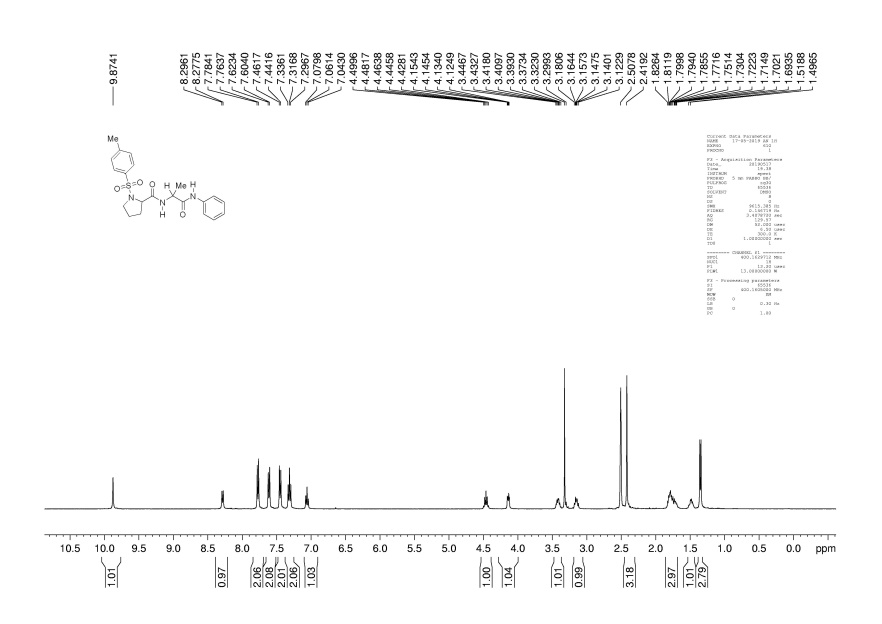


^1^H NMR of **9a**


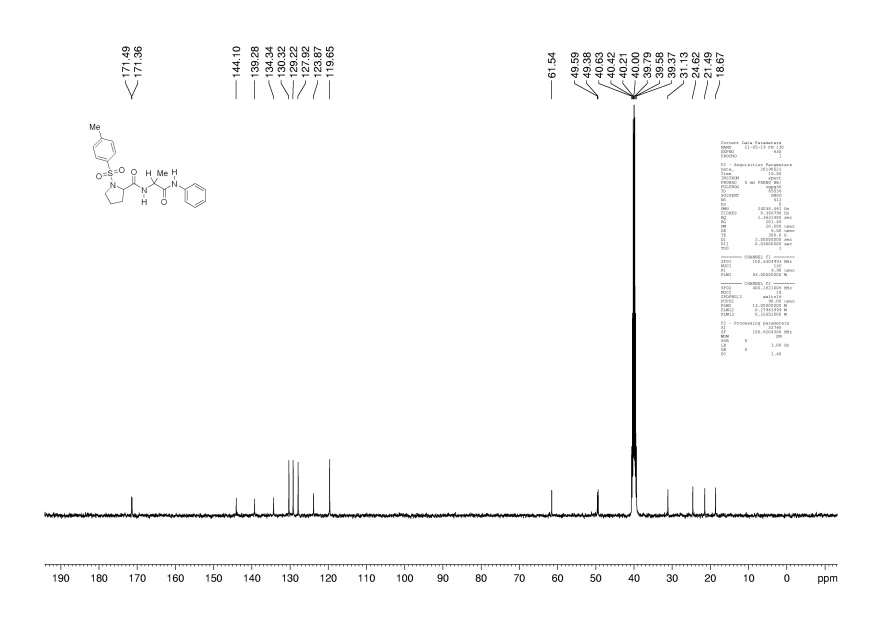


^13^C NMR of **9a**

HRMS of **9a**

**
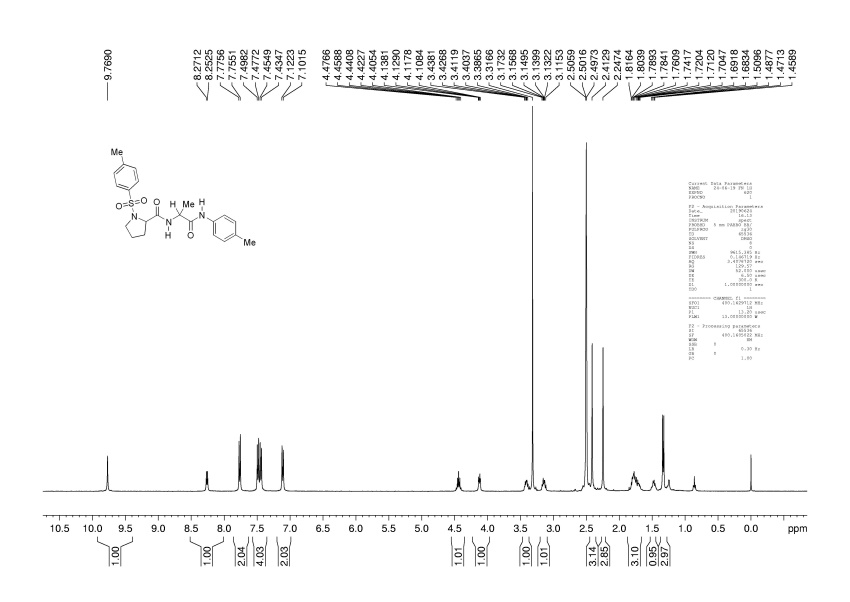
**

^1^H NMR of **9b**

**
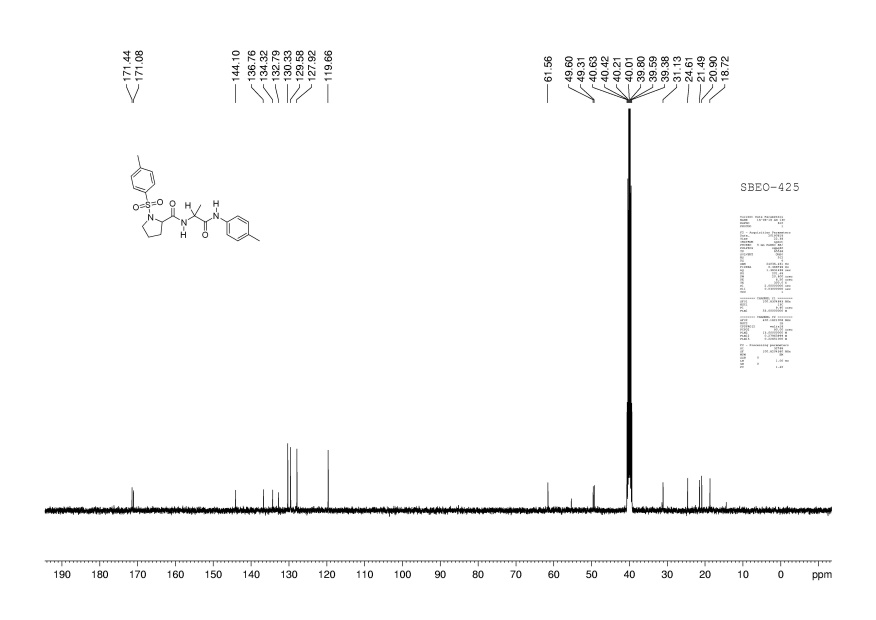
**

^13^C NMR of **9b**

HRMS of **9b**

**
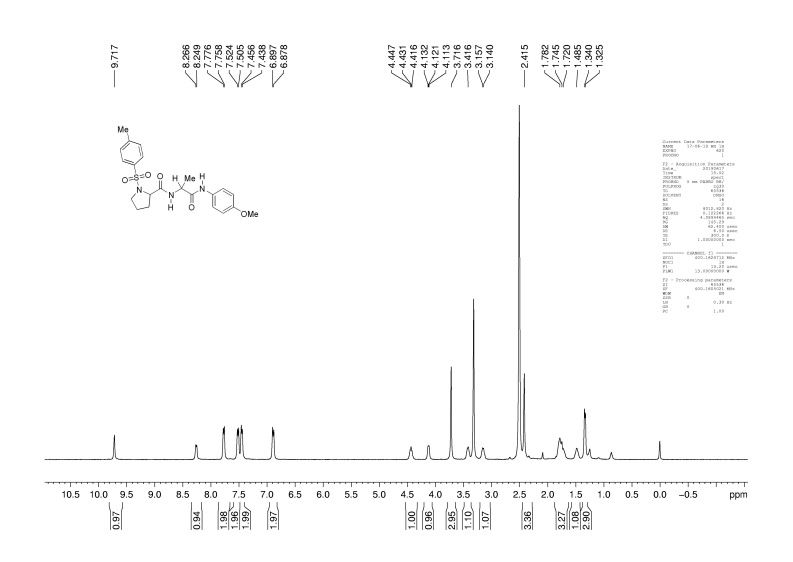
**

^1^H NMR of **9c**

**
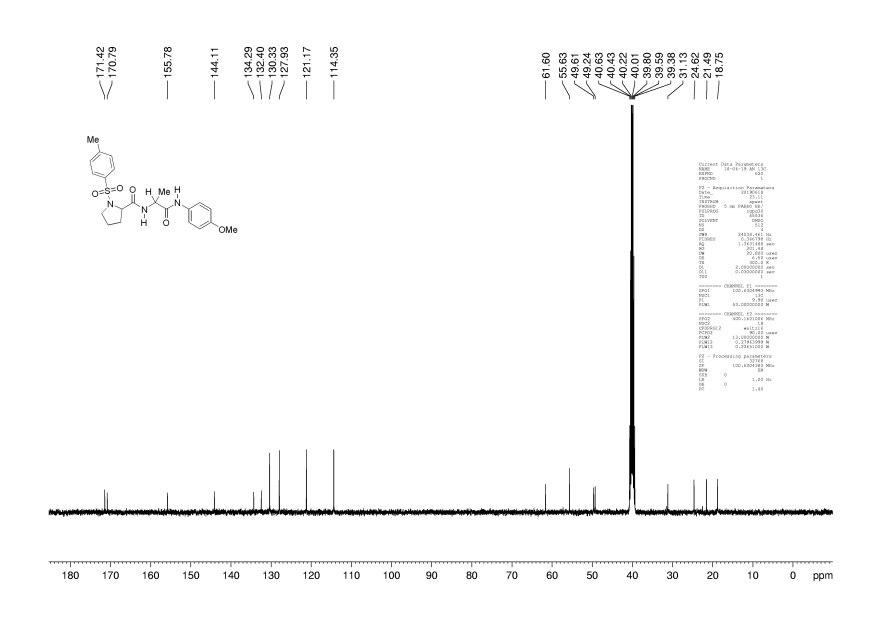
**

^13^C NMR of **9c**

HRMS of **9c**

**
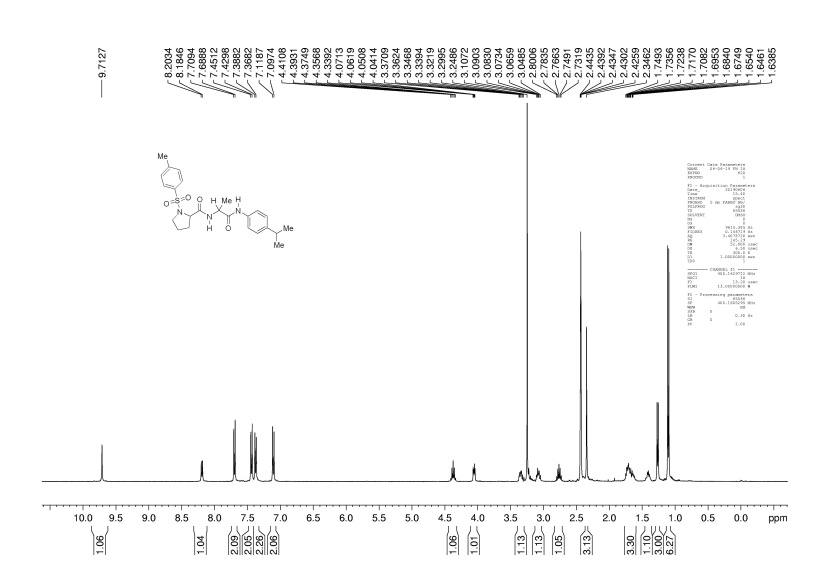
**

^1^H NMR of **9d**


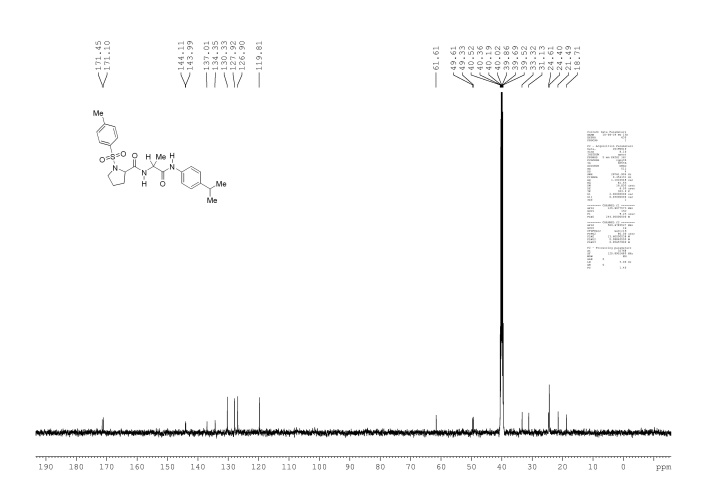


^13^C NMR of **9d**

HRMS of **9d**

**
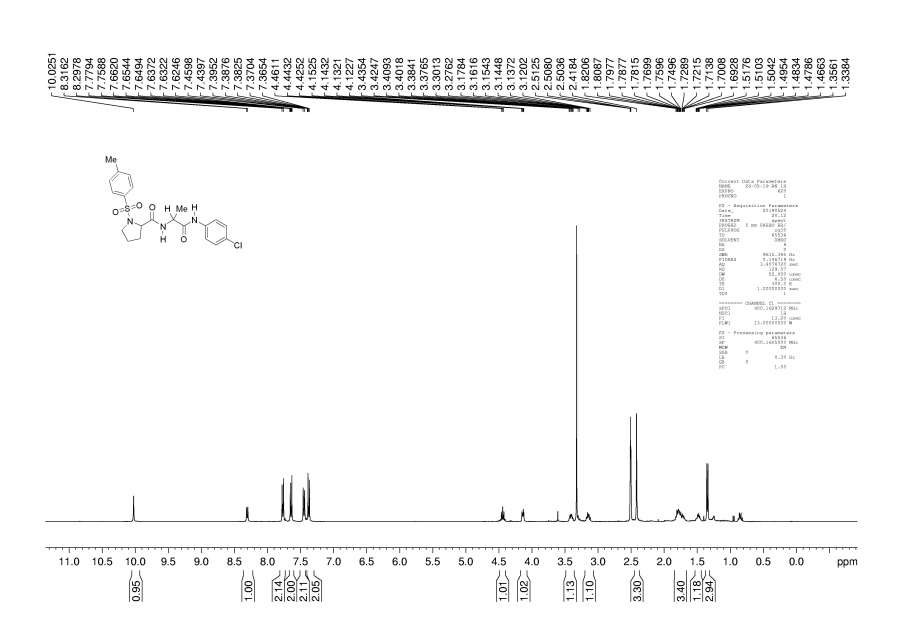
**

^1^H NMR of **9e**

**
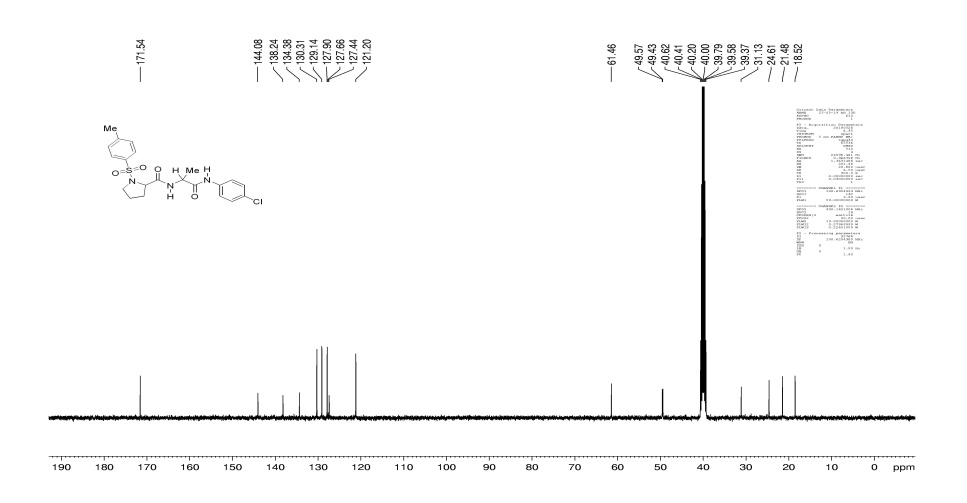
**

^13^C NMR of **9e**

HRMS of **9e**

**
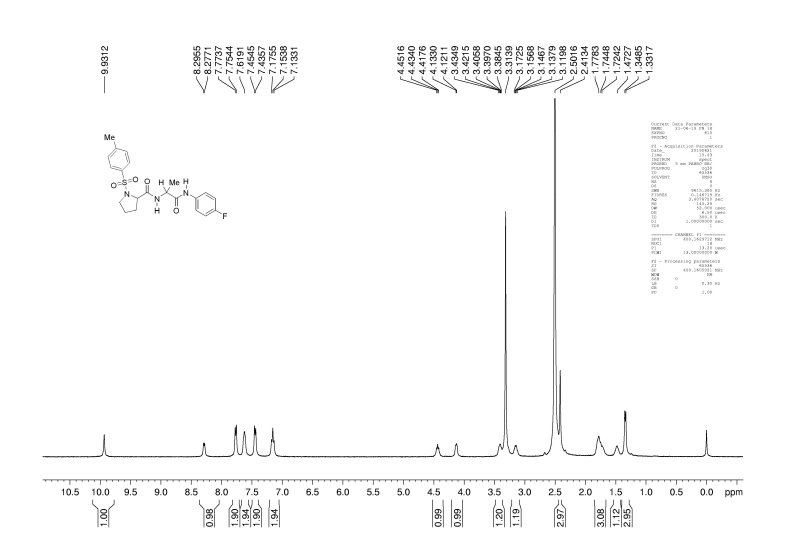
**

^1^H NMR of **9f**

**
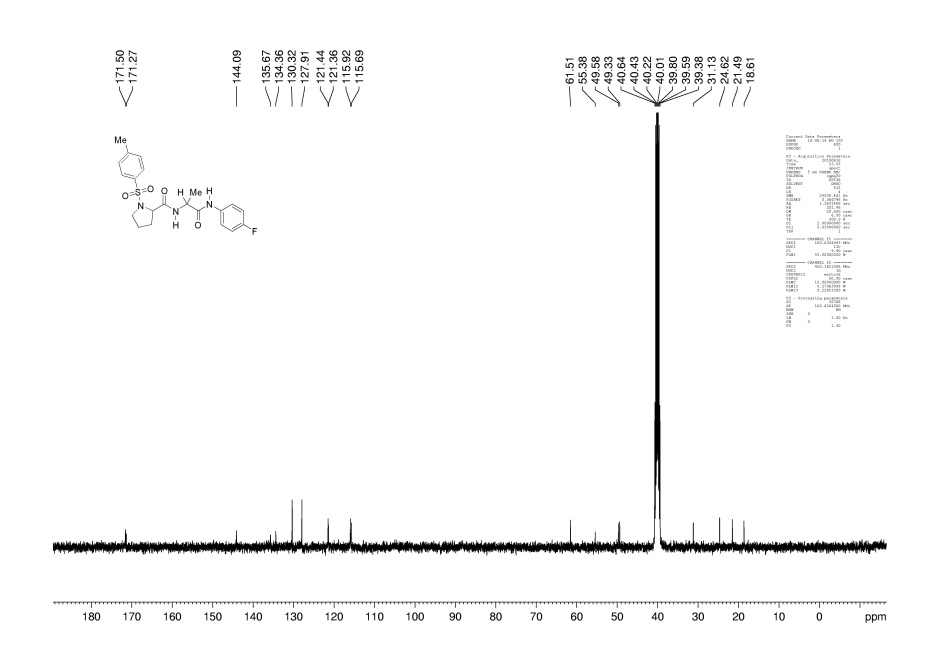
**

^13^C NMR of **9f**

HRMS of **9f**

**
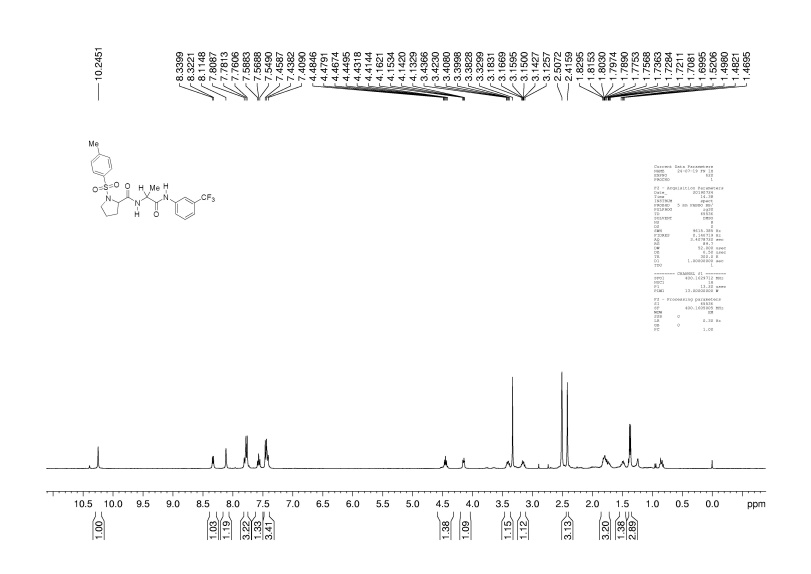
**

^1^H NMR of **9g**

**
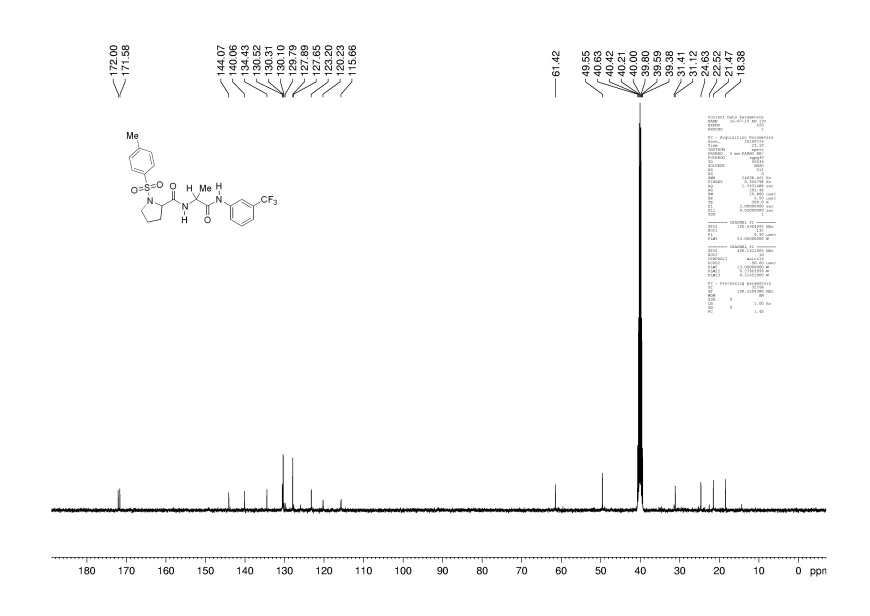
**

^13^C NMR of **9g**

HRMS of **9g**

**
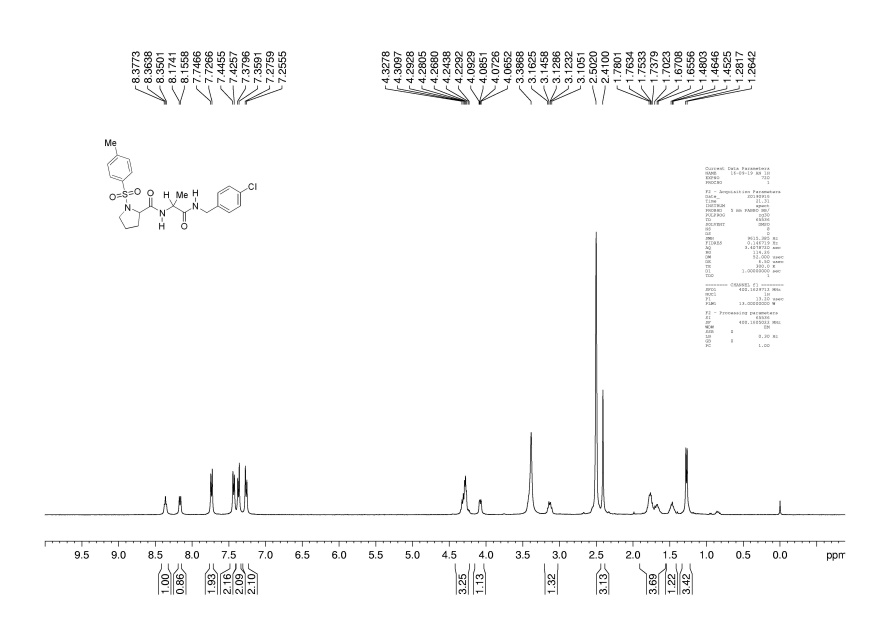
**

^1^H NMR of **9h**

**
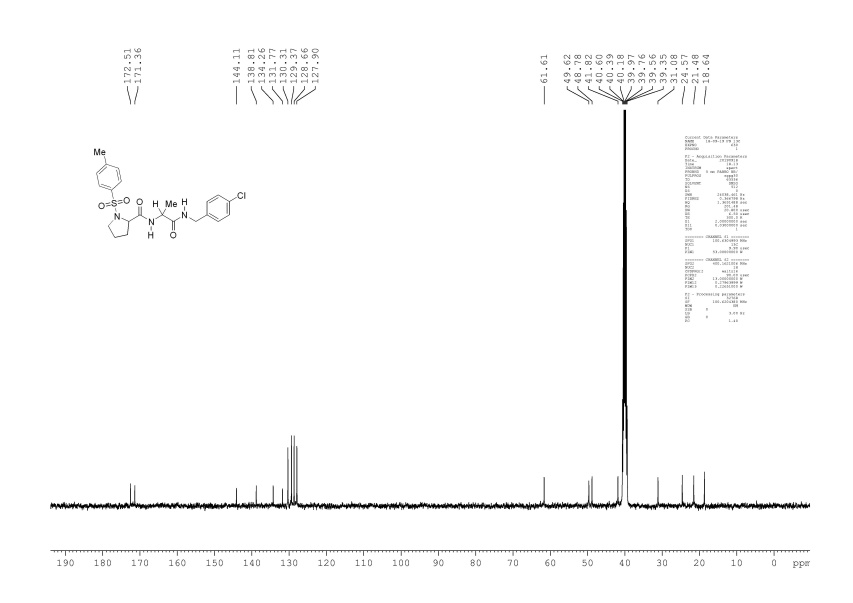
**

^13^C NMR of **9h**

HRMS of **9h**


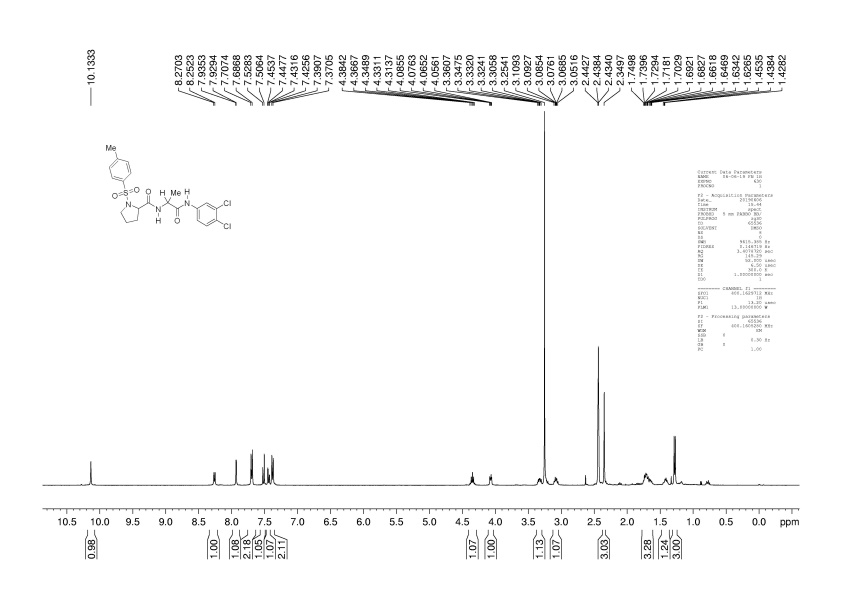


^1^H NMR of **9i**

**
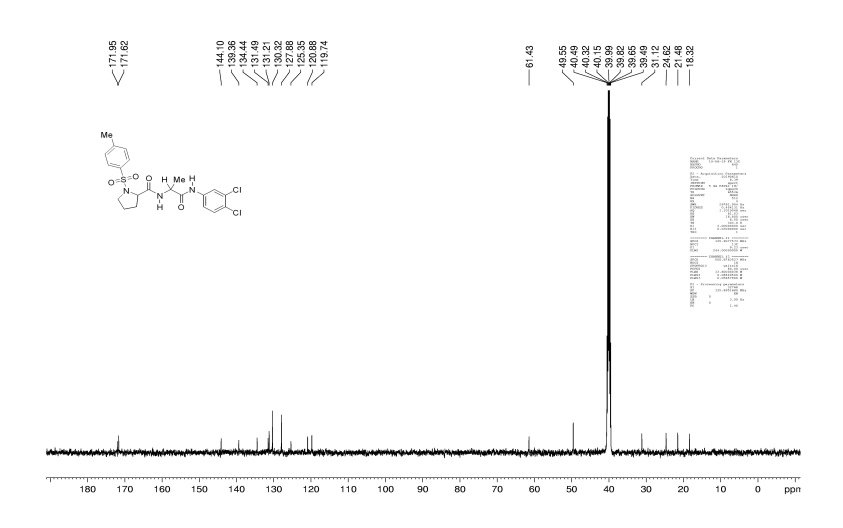
**

^13^C NMR of **9i**

HRMS of **9i**

**
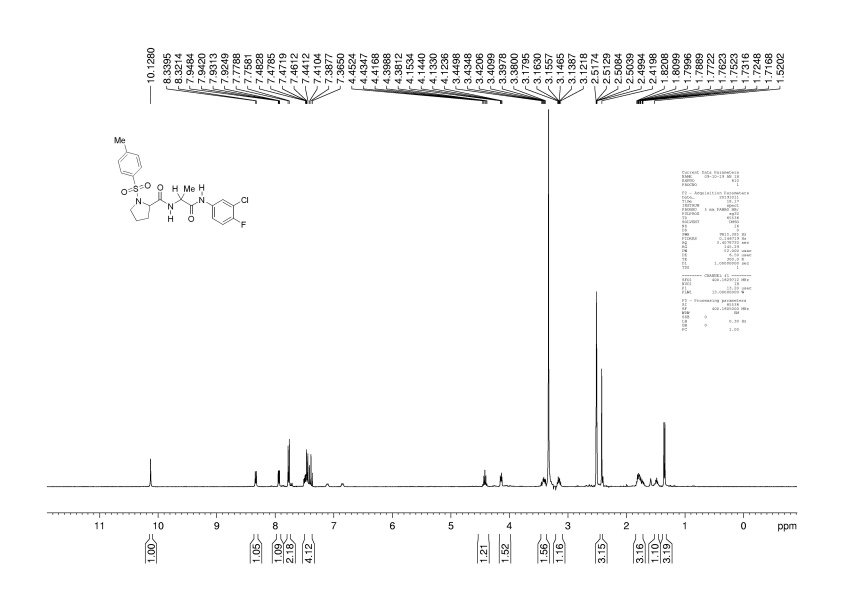
**

^1^H NMR of **9j**

**
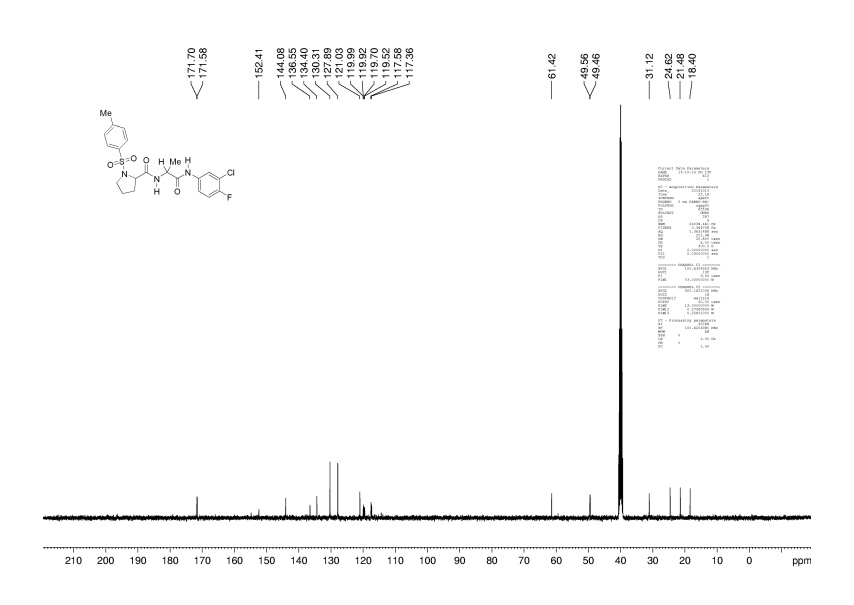
**

^13^C NMR of **9j**

HRMS of **9j**

**
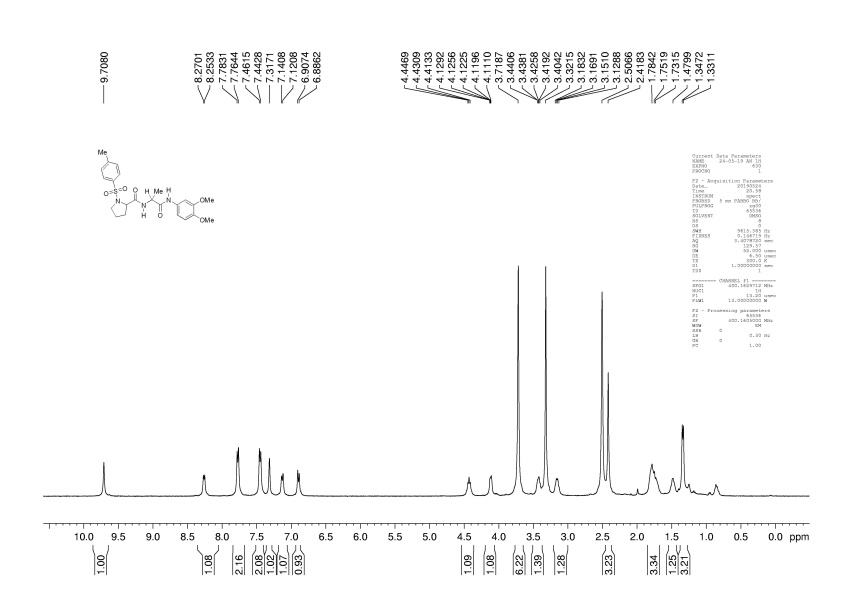
**

^1^H NMR of **9k**

**
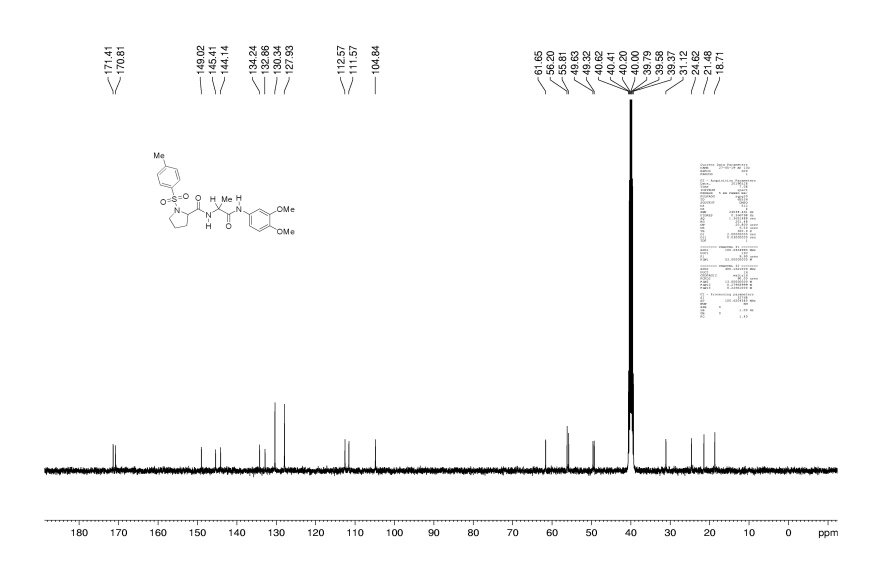
**

^13^C NMR of **9k**

HRMS of **9k**

**
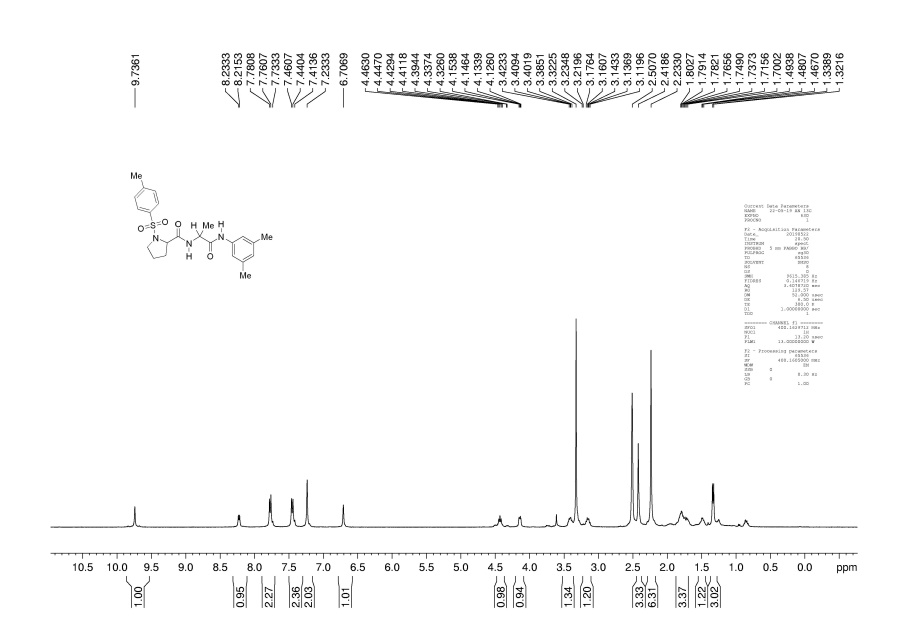
**

^1^H NMR of **9l**

**
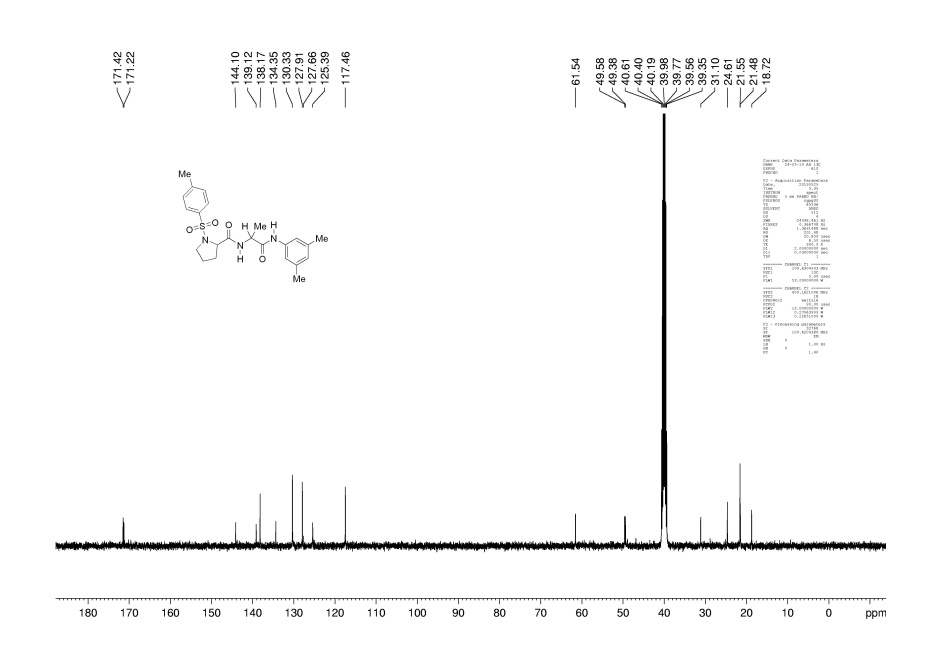
**

^13^C NMR of **9l**

HRMS of **9l**

**
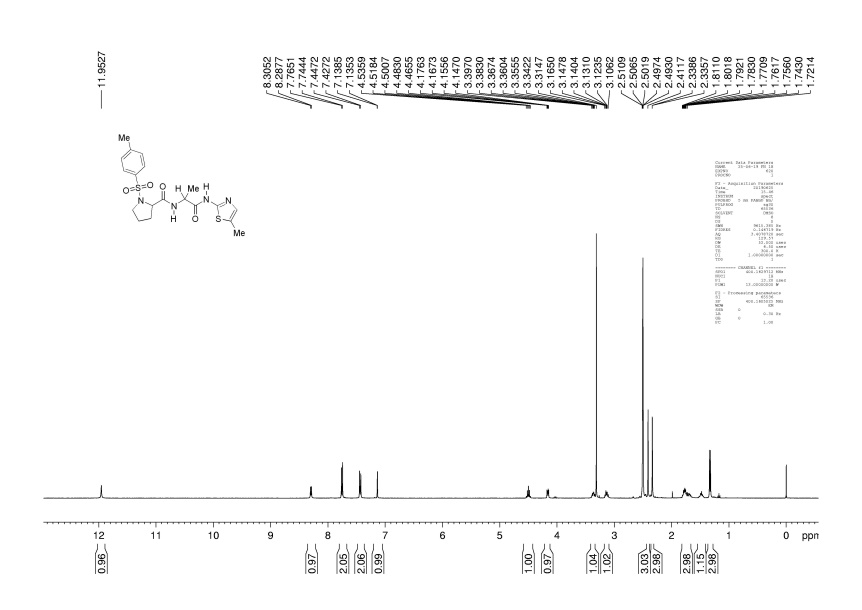
**

^1^H NMR of **9m**

**
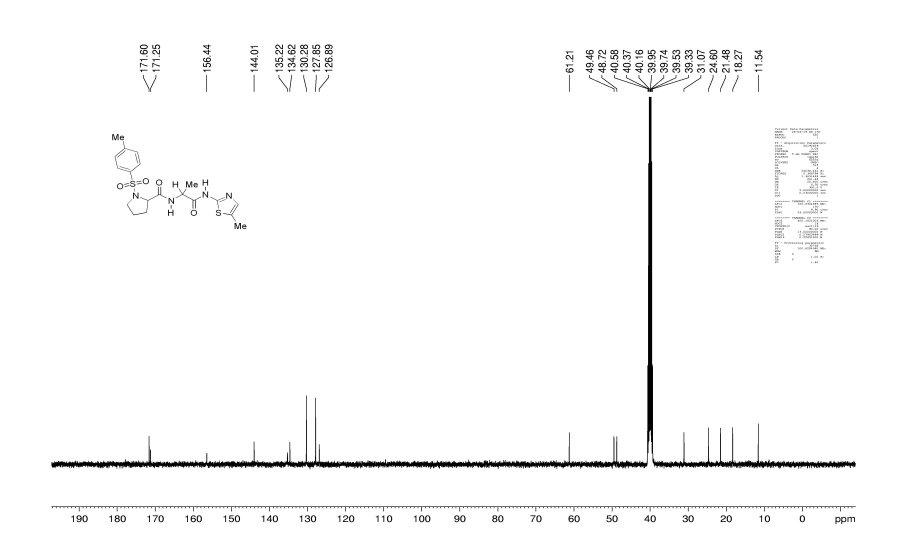
**

^13^C NMR of **9m**

HRMS of **9m**

**
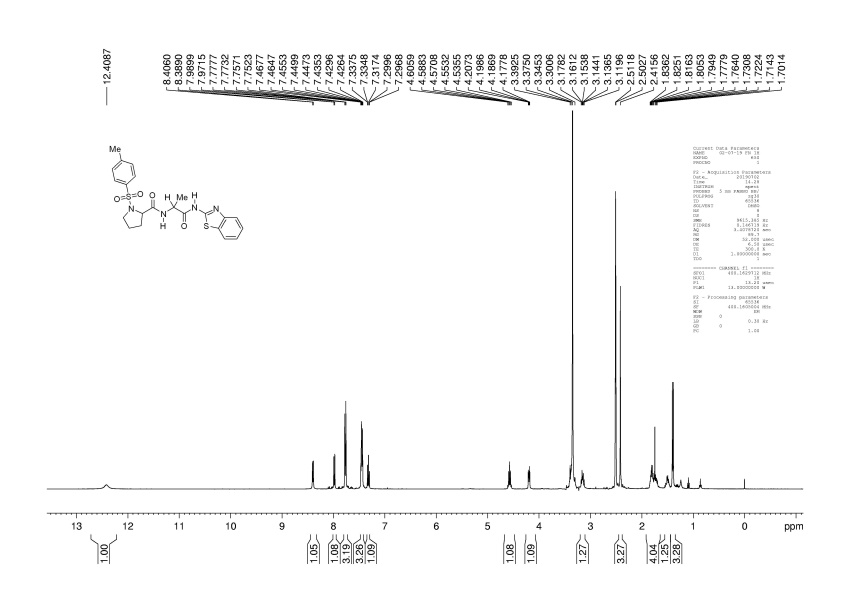
**

^1^H NMR of **9n**

**
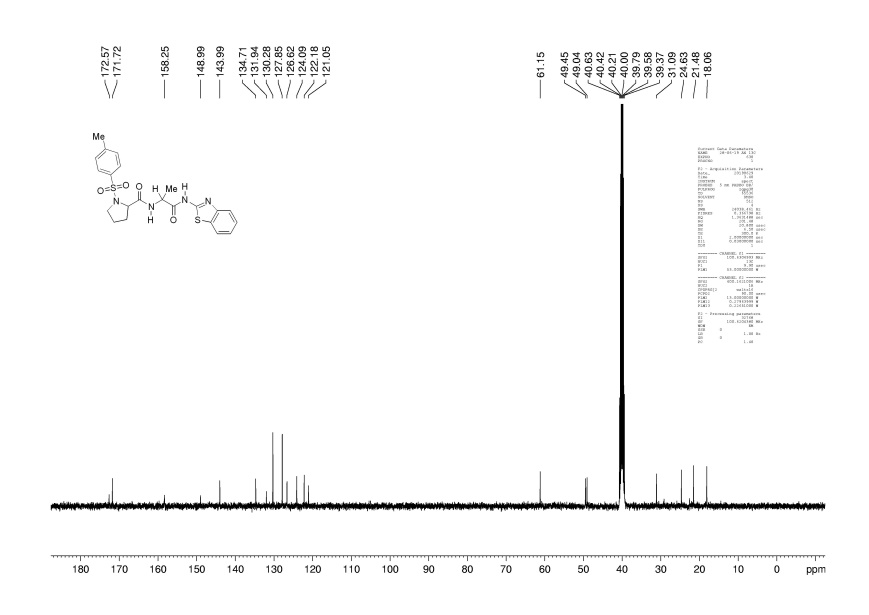
**

^13^C NMR of **9n**

HRMS of **9n**

**
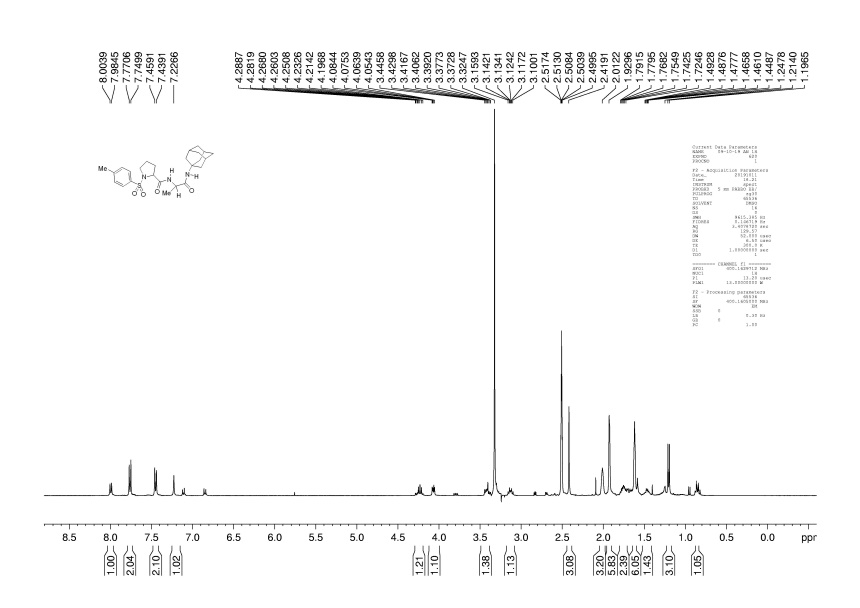
**

^1^H NMR of **9o**

**
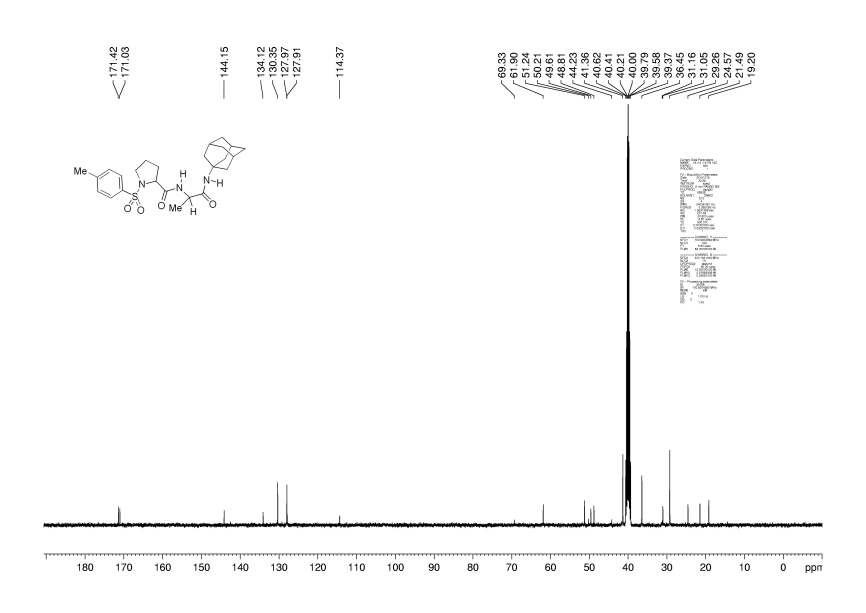
**

^13^C NMR of **9o**

HRMS of **9o**

**
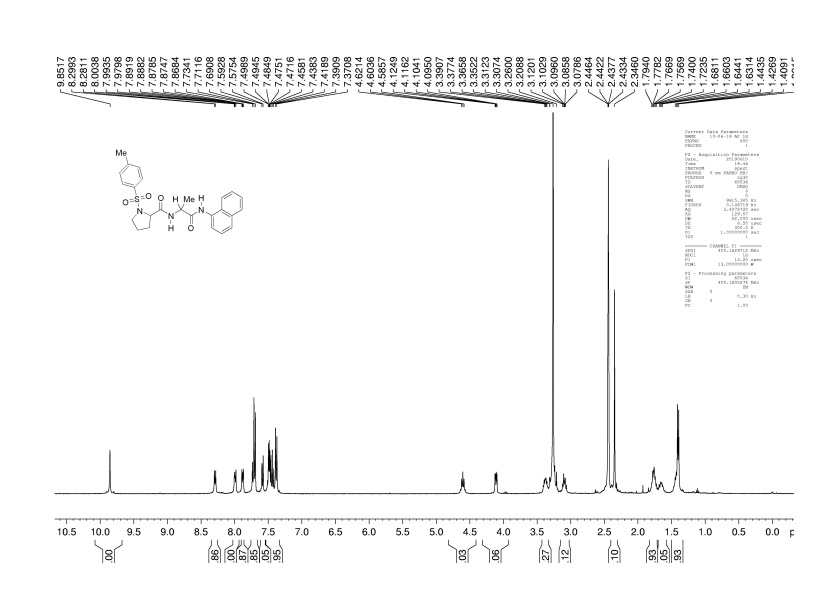
**

^1^H NMR of **9p**

**
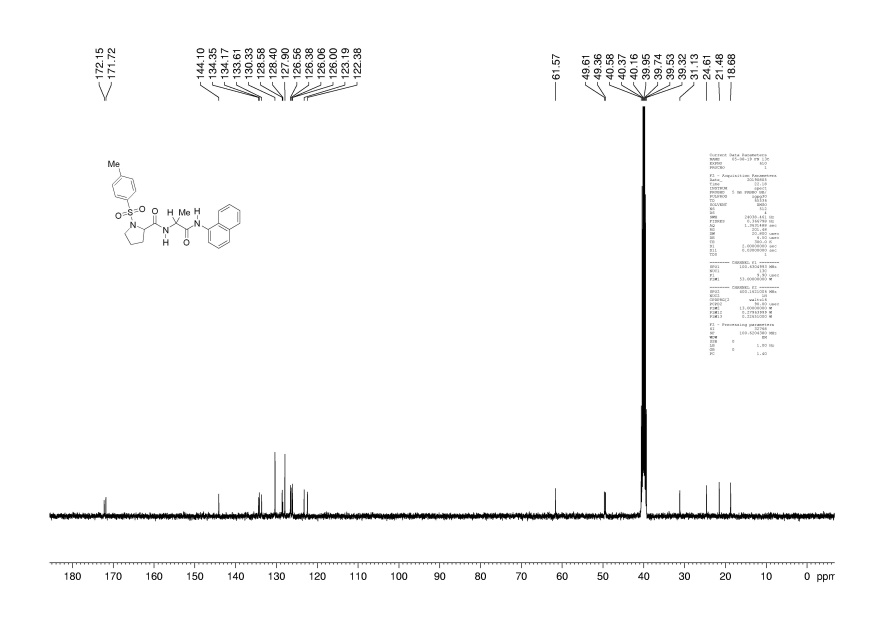
**

^13^C NMR of **9p**

HRMS of **9p**

**
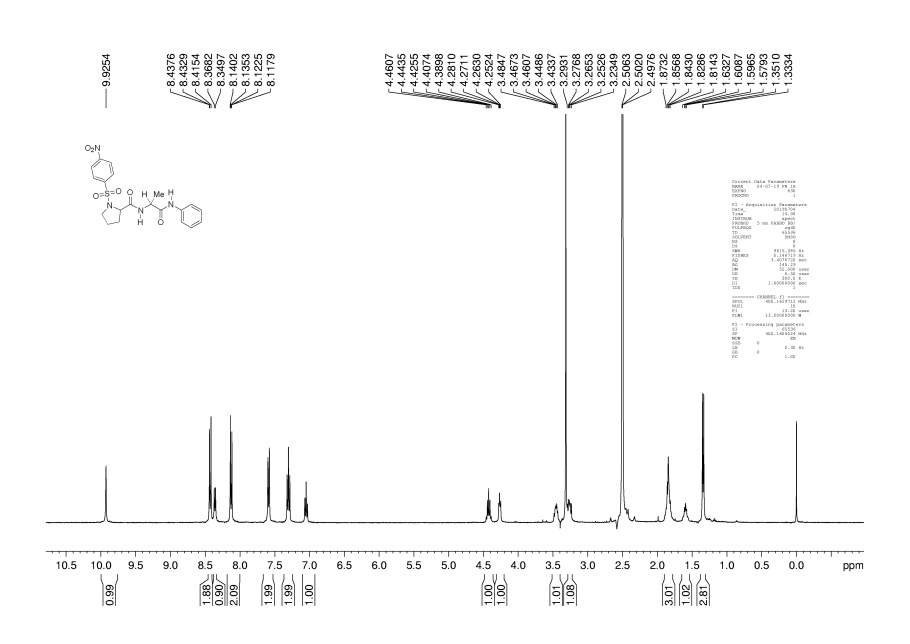
**

^1^H NMR of **10a**

**
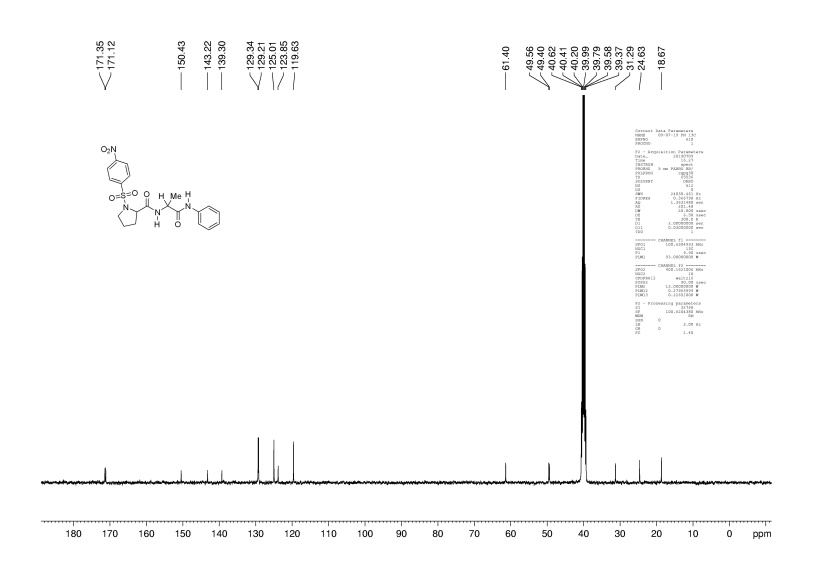
**

^13^C NMR of **10a**

HRMS of **10a**


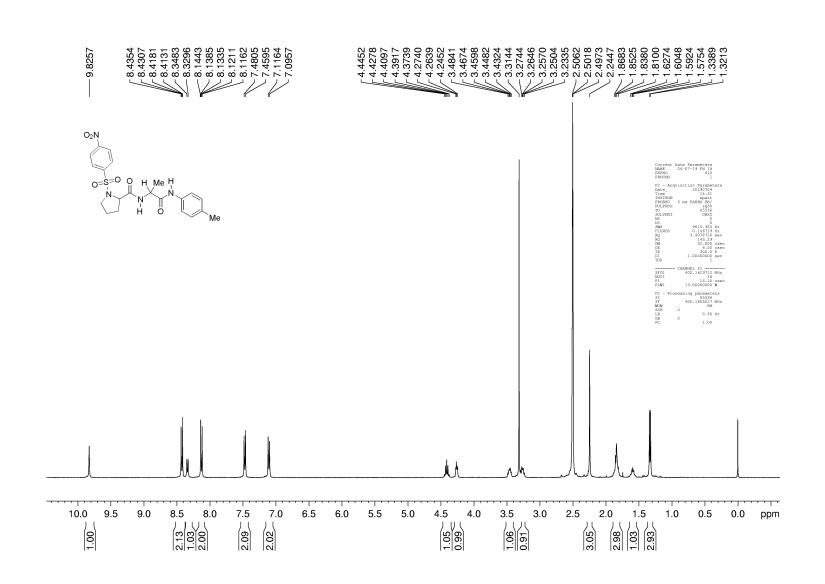


^1^H NMR of **10b**

**
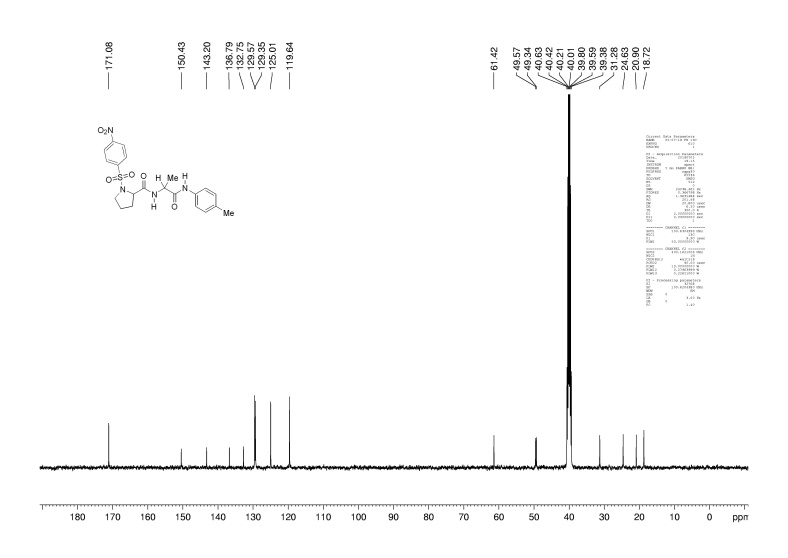
**

^13^C NMR of **10b**

HRMS of **10b**

**
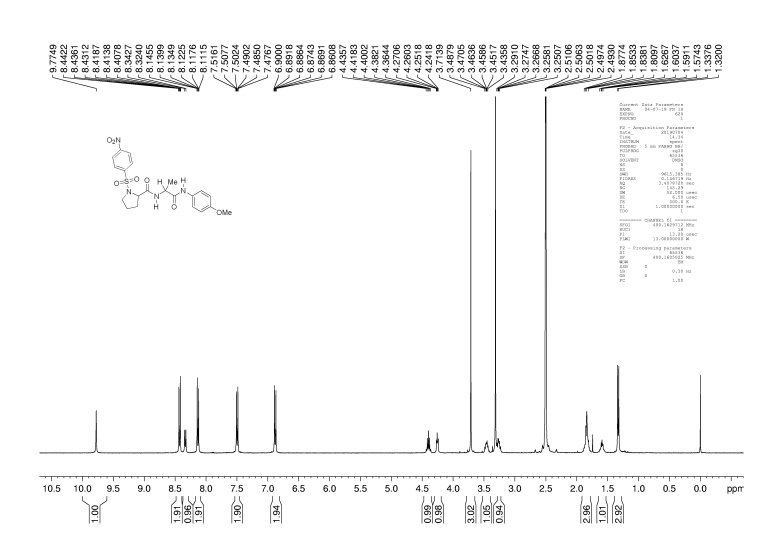
**

^1^H NMR of **10c**

**
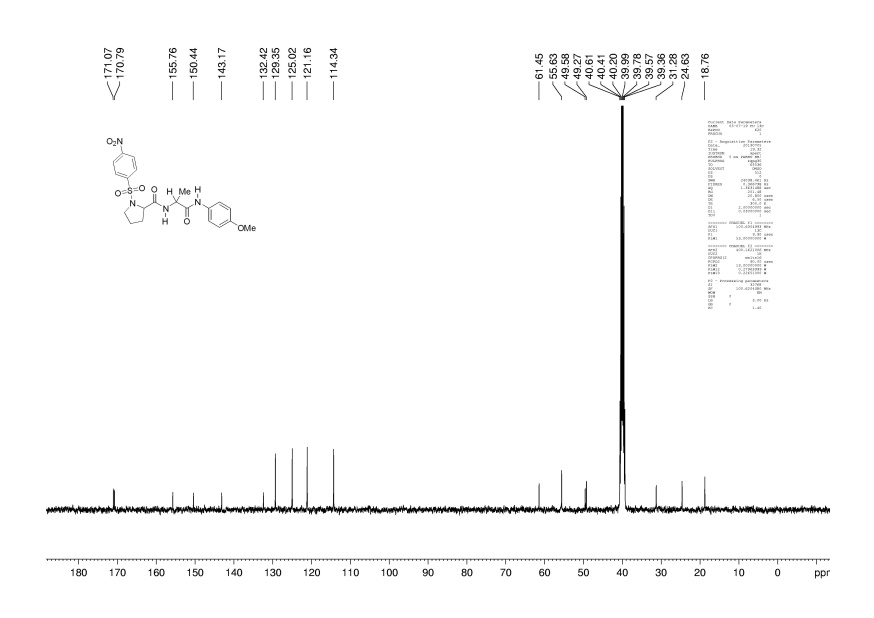
**

^13^C NMR of **10c**

HRMS of **10c**


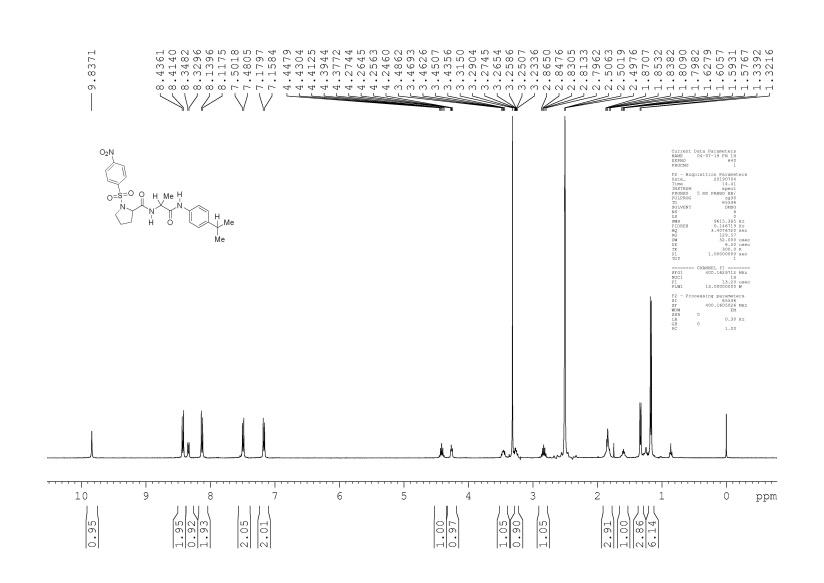


^1^H NMR of **10d**

**
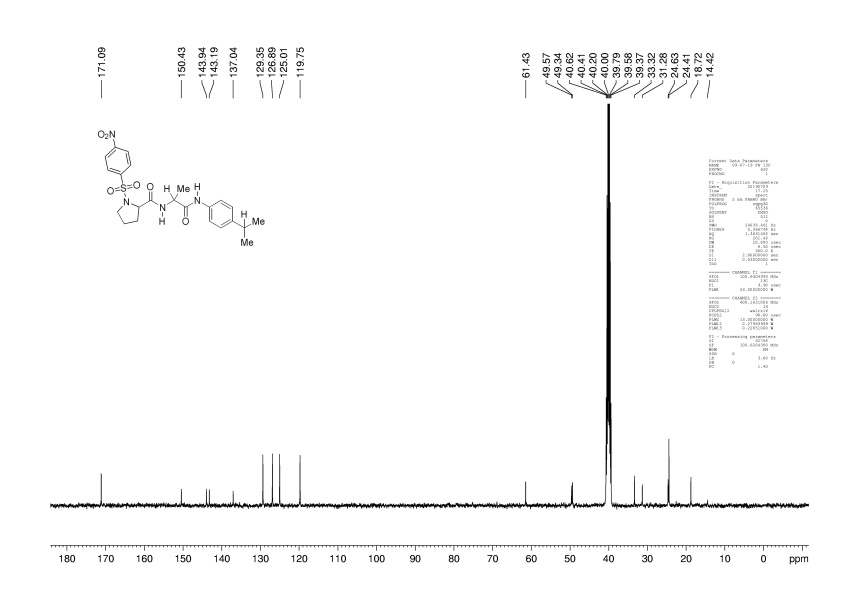
**

^13^C NMR of **10d**

HRMS of **10d**

**
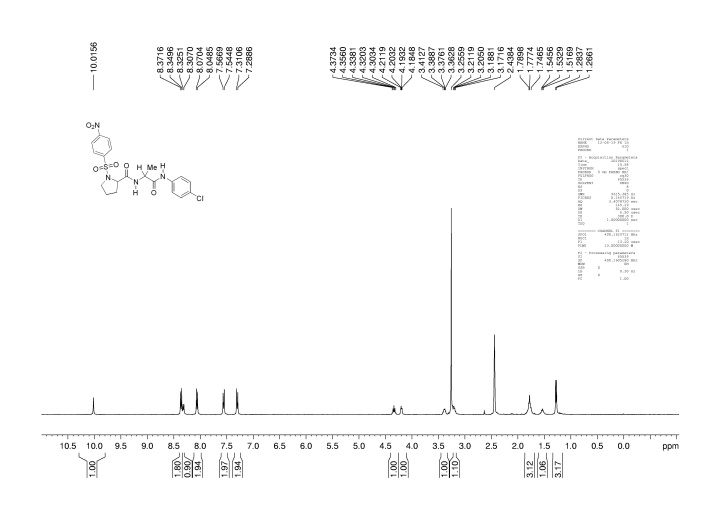
**

^1^H NMR of **10e**


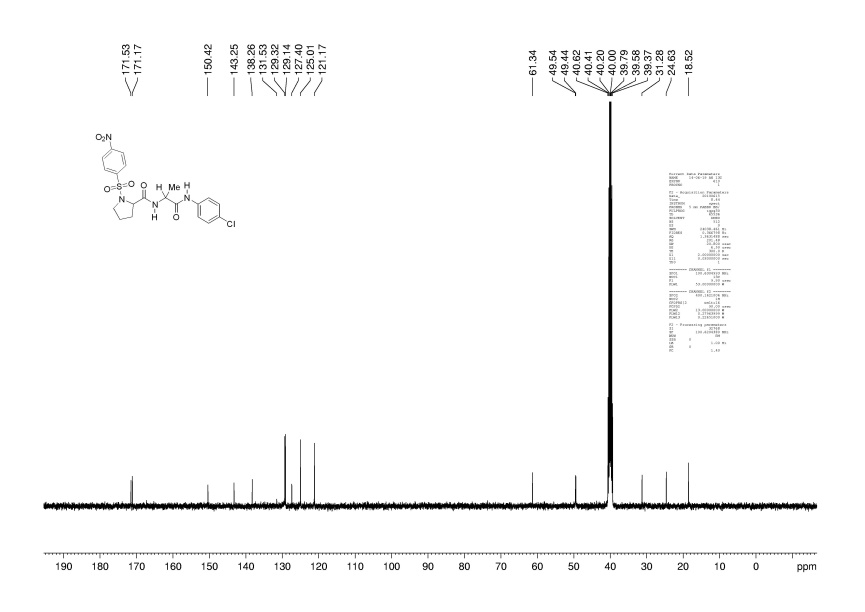


^13^C NMR of **10e**

HRMS of **10e**

**
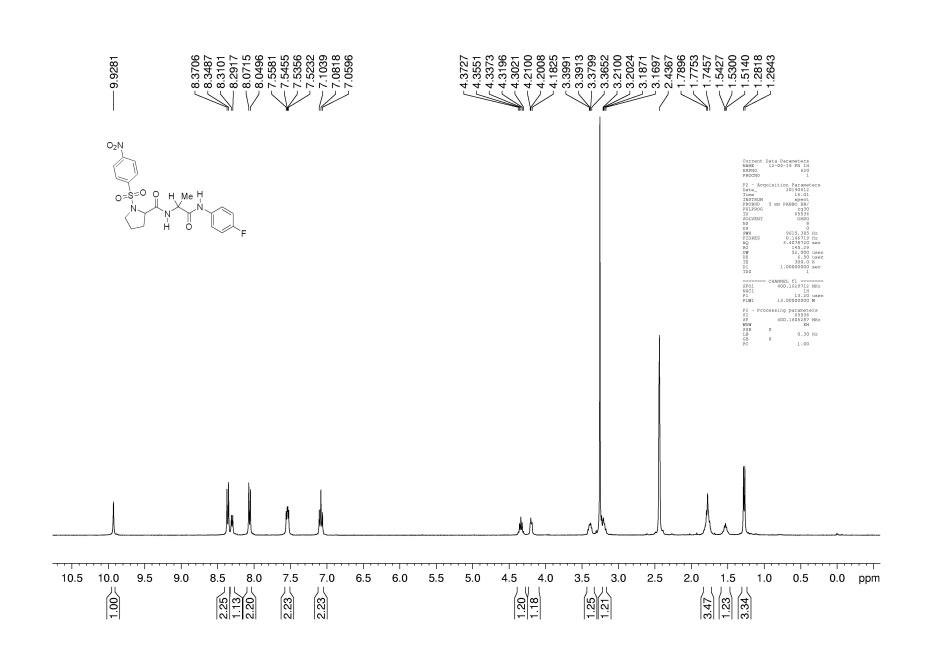
**

^1^H NMR of **10f**

**
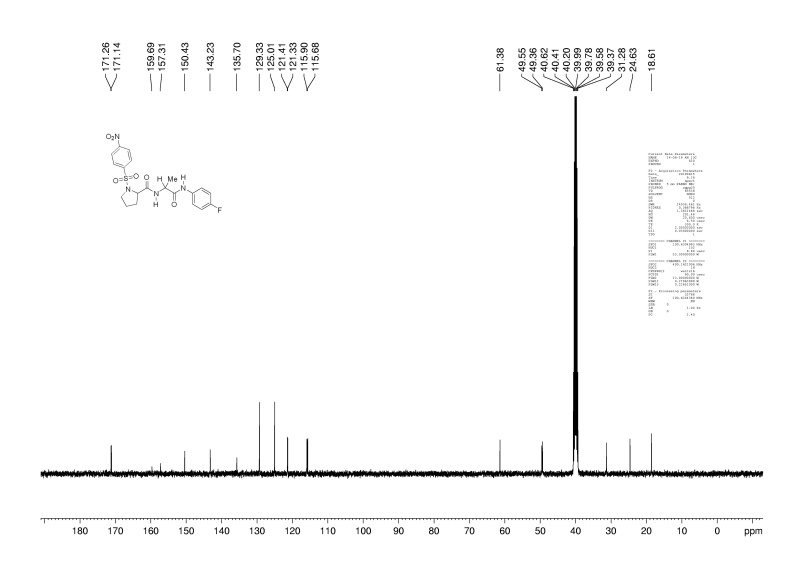
**

^13^C NMR of **10e**

HRMS of **10e**

**
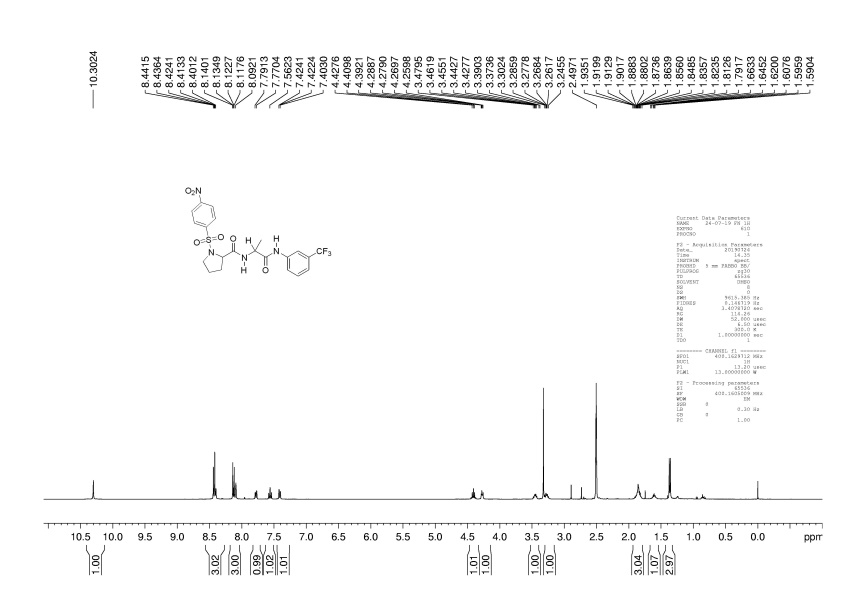
**

^1^H NMR of **10g**

**
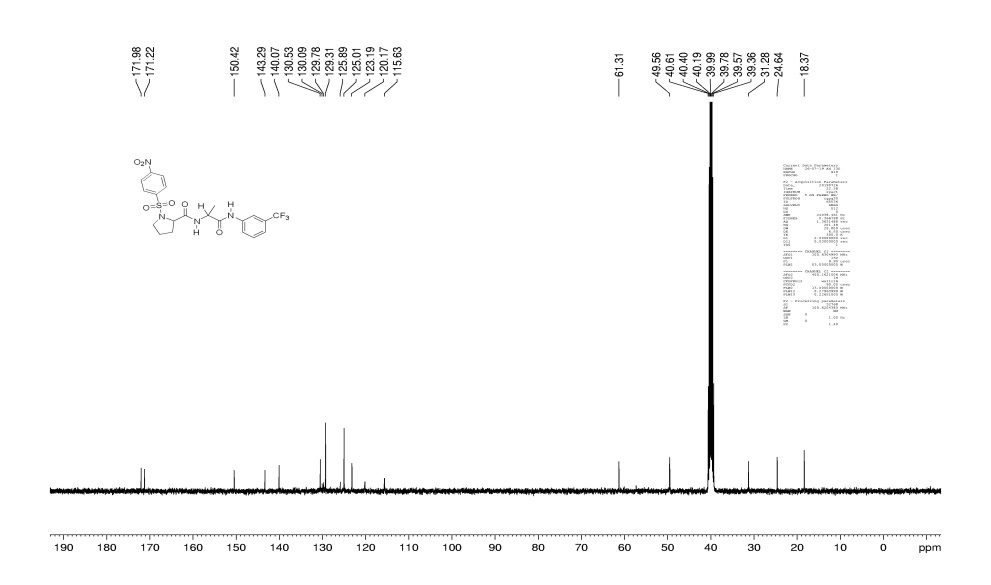
**

^13^C NMR of **10g**

HRMS of **10g**

**
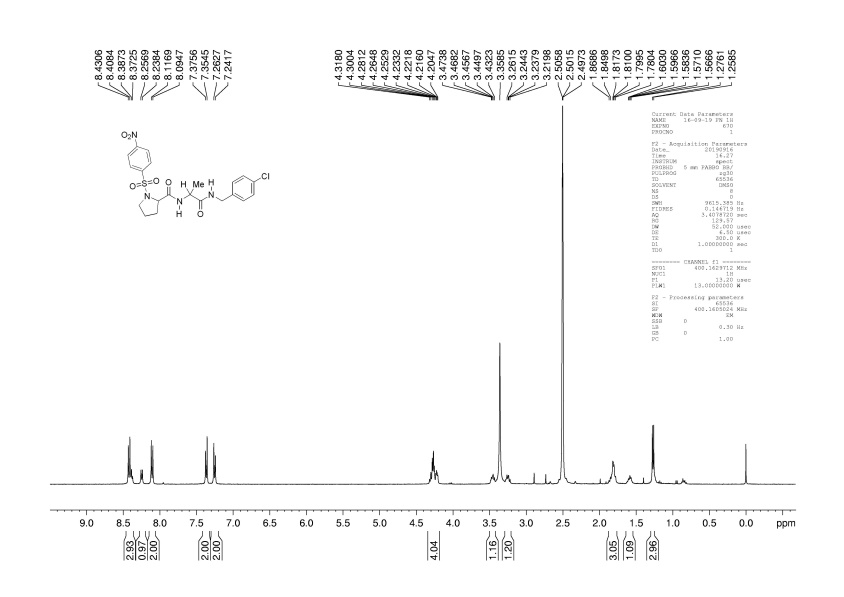
**

^1^H NMR of **10h**

**
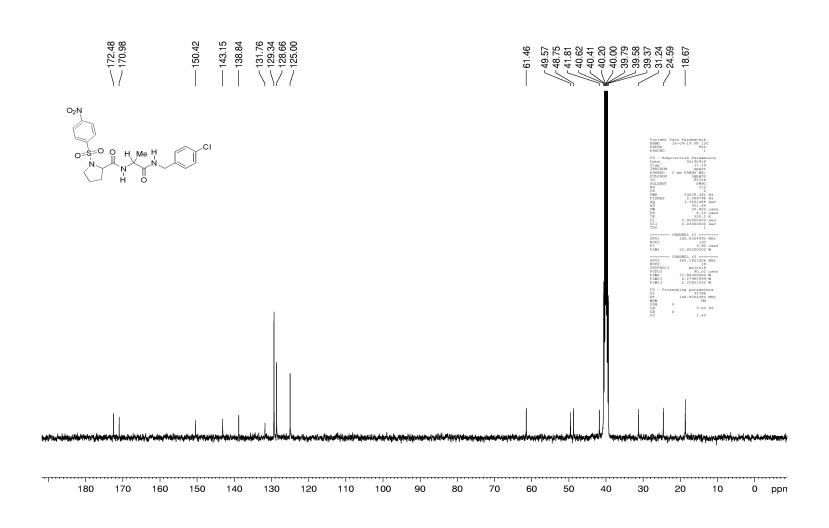
**

^13^C NMR of **10h**

HRMS of **10h**

**
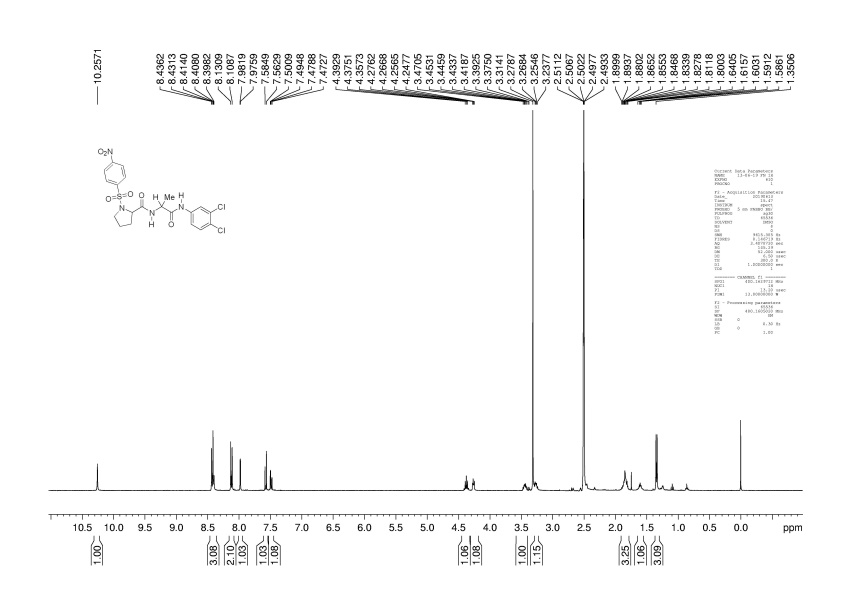
**

^1^H NMR of **10i**

**
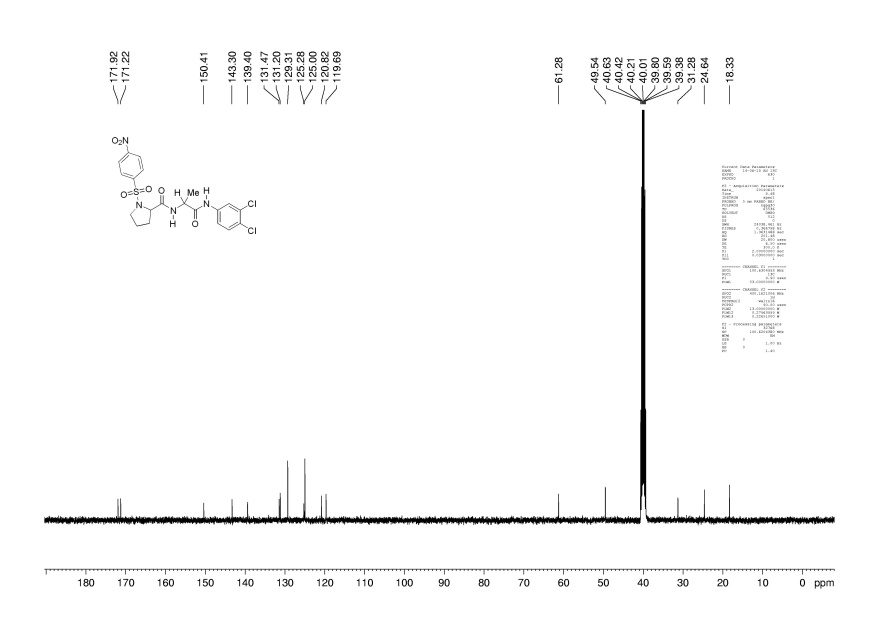
**

^13^C NMR of **10i**

HRMS of **10i**

**
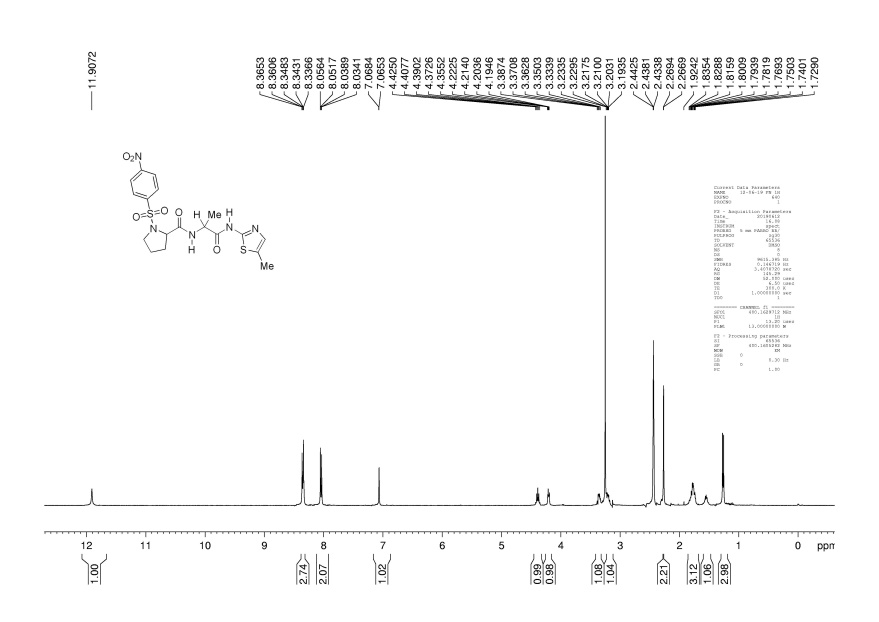
**

^1^H NMR of **10j**

**
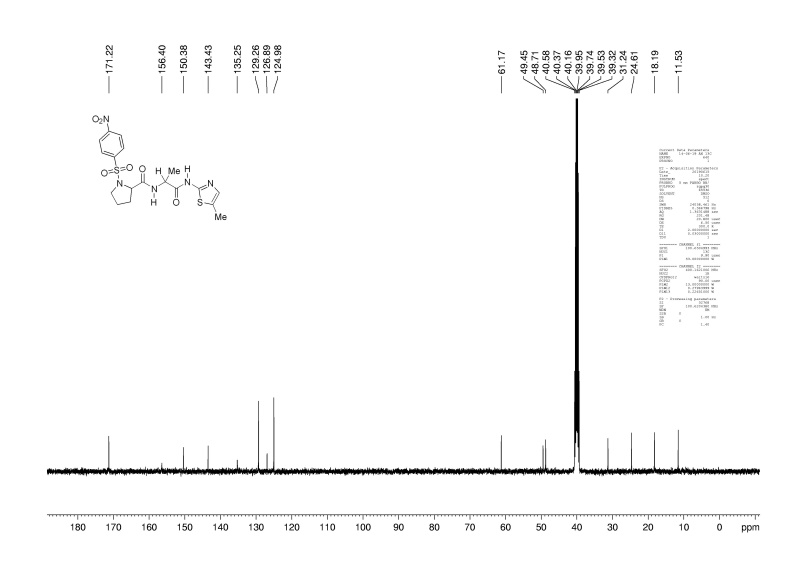
**

^13^C NMR of **10j**

HRMS of **10j**


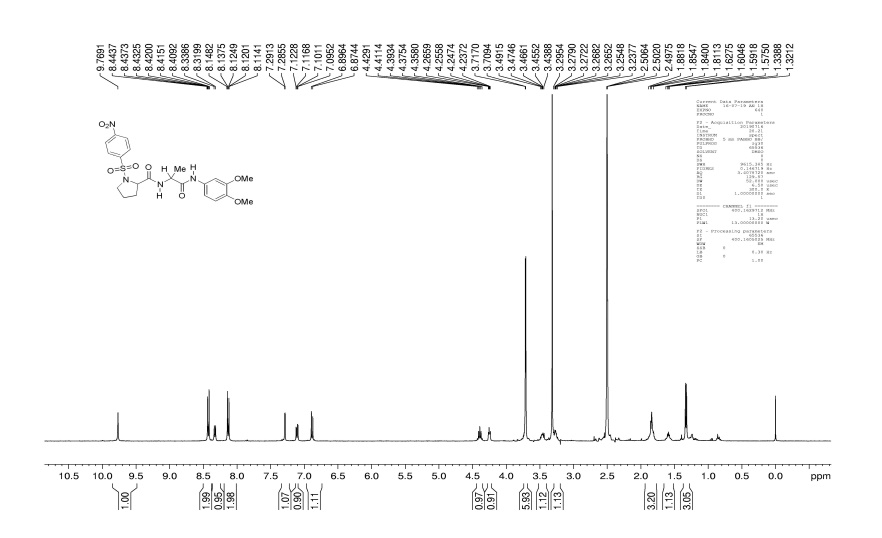


^1^H NMR of **10k**

**
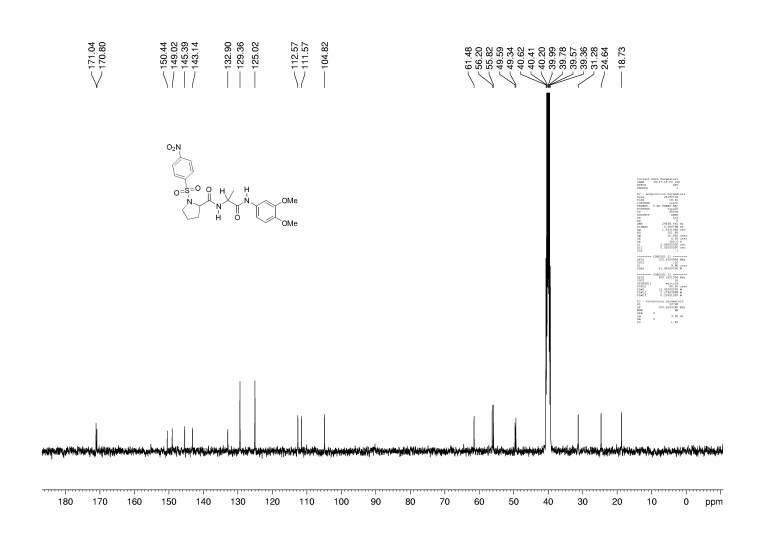
**

^13^C NMR of **10k**

HRMS of **10k**

**
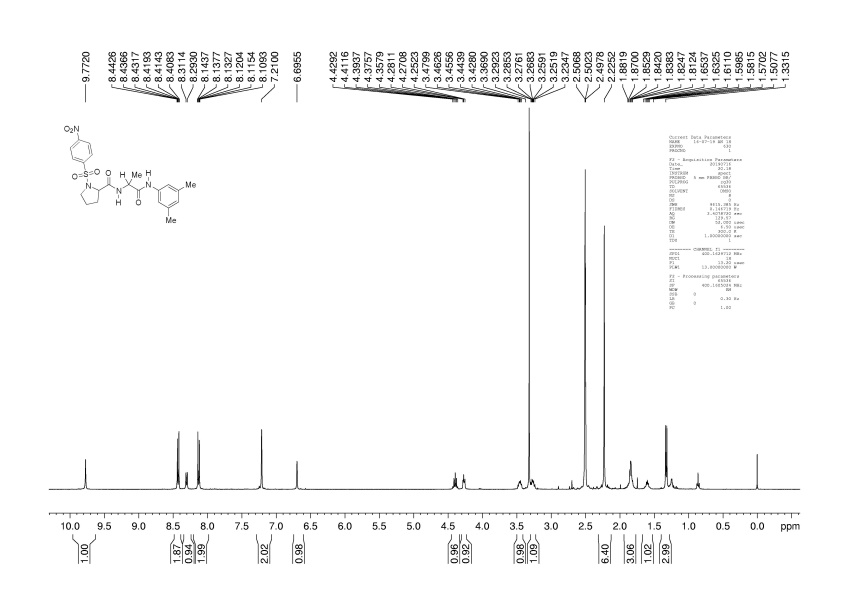
**

^1^H NMR of **10l**

**
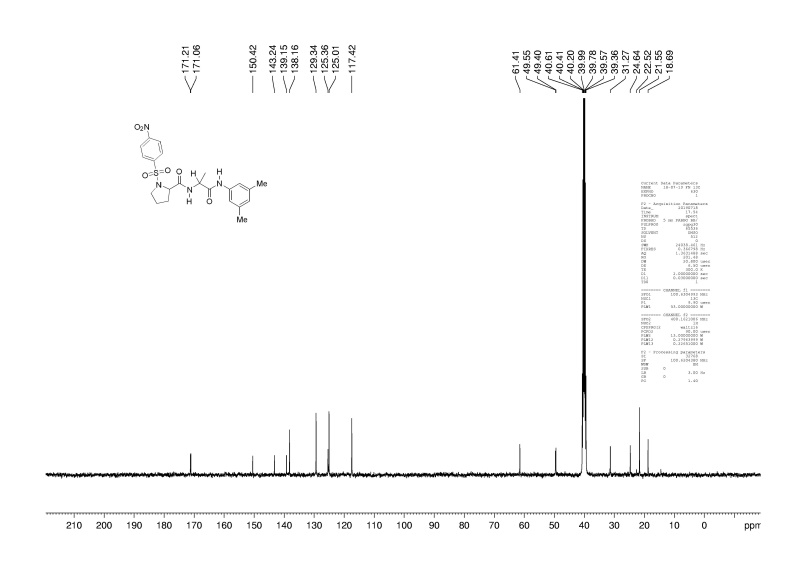
**

^13^C NMR of **10l**

HRMS of **10l**

**
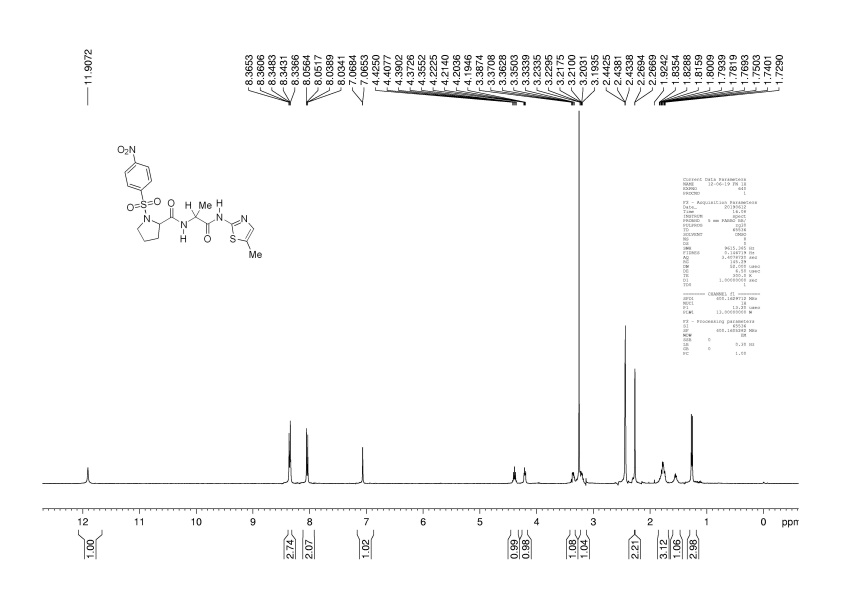
**

^1^H NMR of **10m**

**
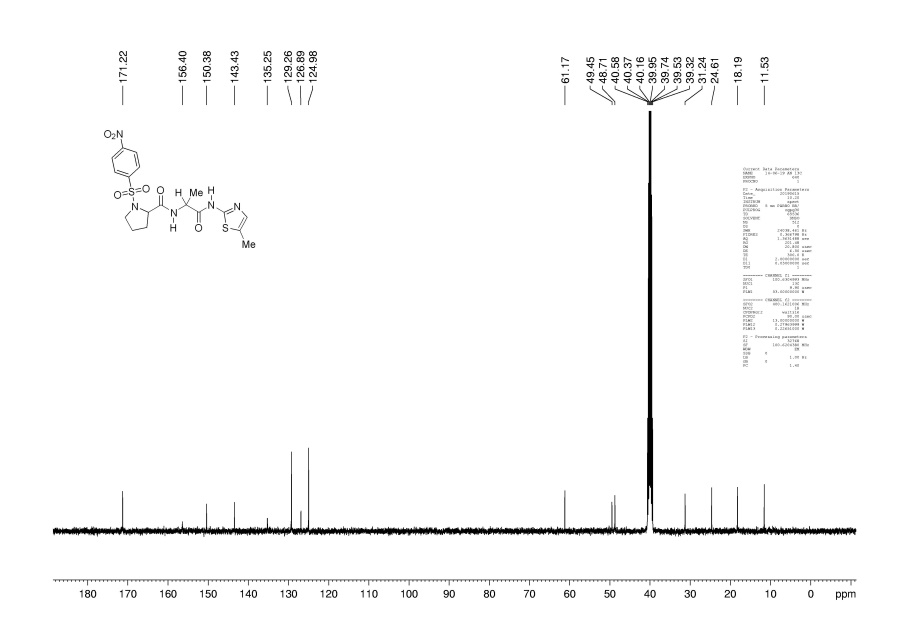
**

^13^C NMR of **10m**

HRMS of **10m**

**
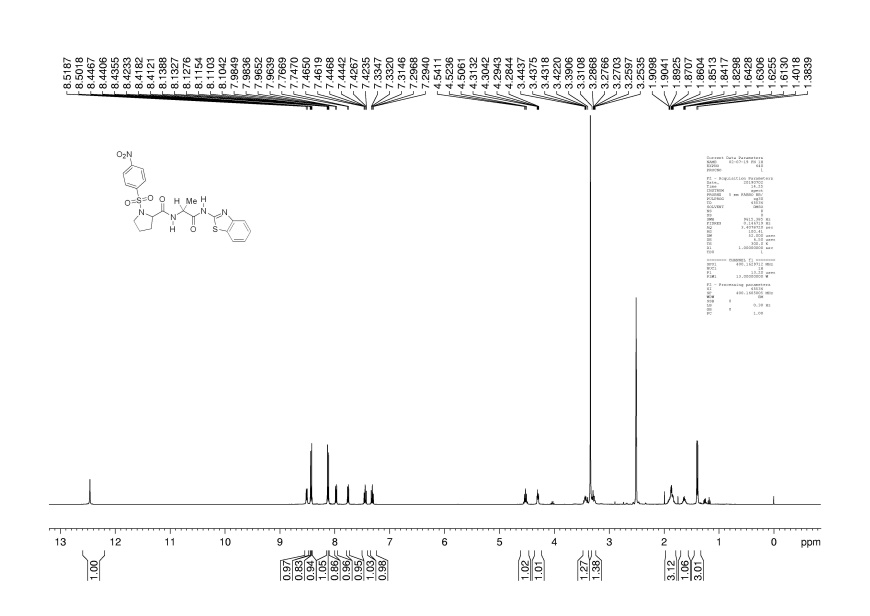
**

^1^H NMR of **10n**

**
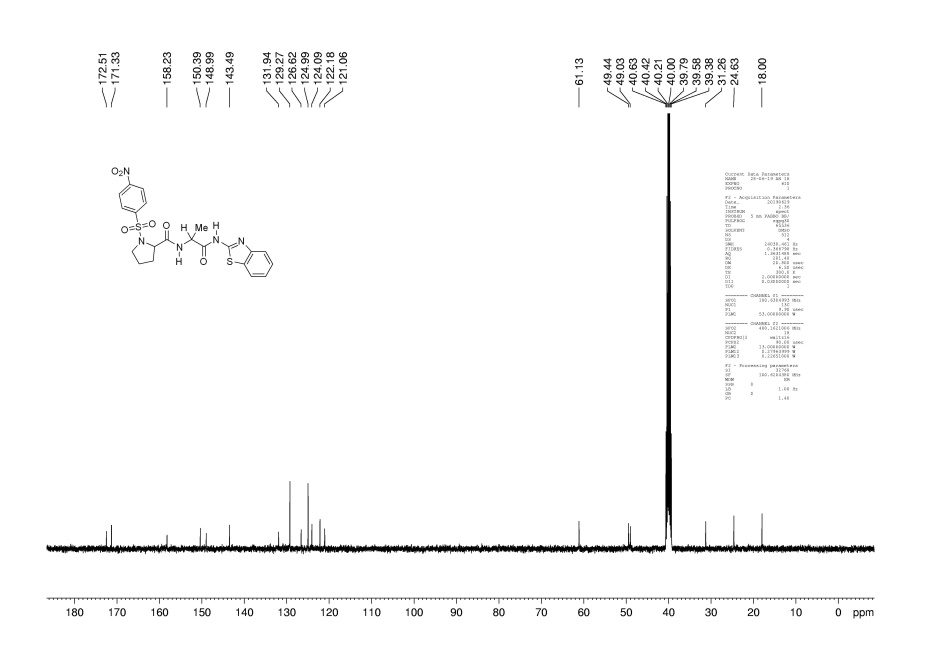
**

^13^C NMR of **10n**

HRMS of **10n**

**
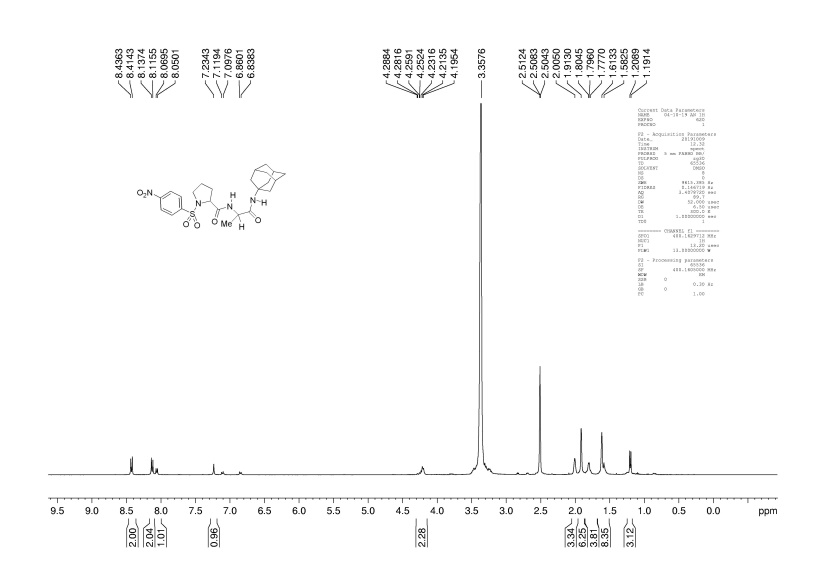
**

^1^H NMR of **10o**


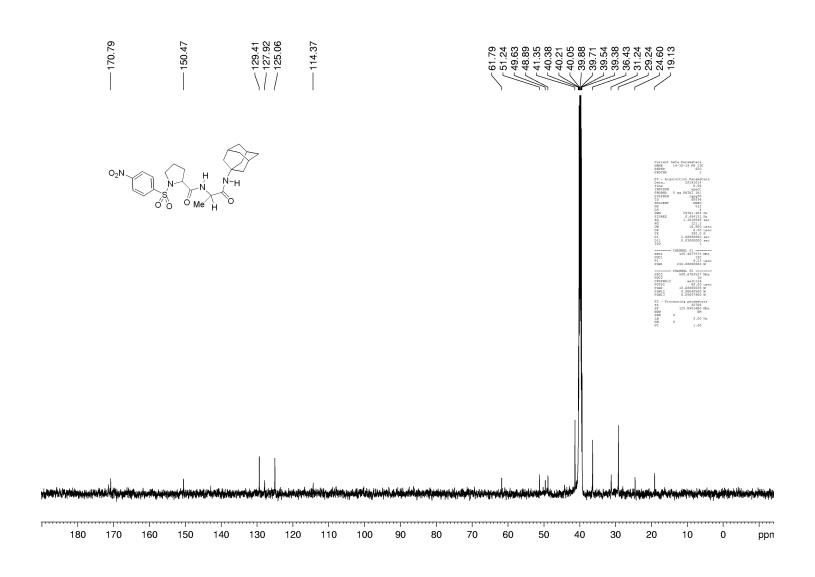


^13^C NMR of **10o**

HRMS of **10o**

**
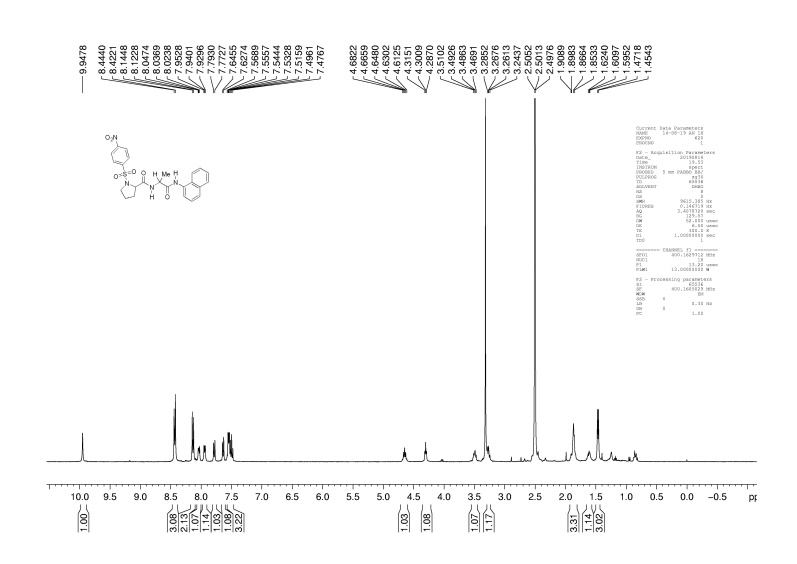
**

^1^H NMR of **10p**

**
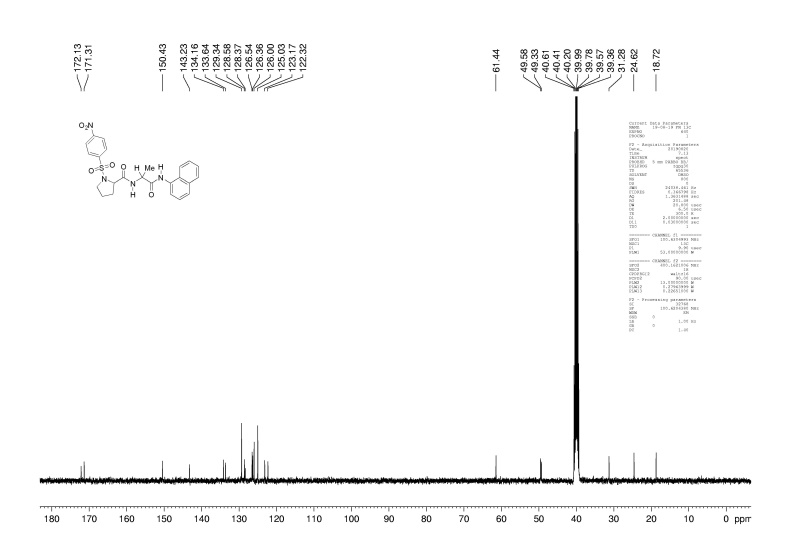
**

^13^C NMR of **10p**

HRMS of **10p**
