# Supplementary material for: New sulphonamide pyrolidine carboxamide derivatives: Synthesis, molecular docking, antiplasmodial and antioxidant activities
Source: PLoS One. 2021 Feb 24;16(2):e0243305. doi: 10.1371/journal.pone.0243305 (PMC7904193; doi:10.1371/journal.pone.0243305)
Supplement: Attachment — Submitted filename: Response to reviewers_Plos One_latest.docx [file pone.0243305.s002.docx]

**REVIEWAL REPORT RESPOND**

**REVIEWER#1** .One the major concern is that all the tested compounds are not very active against malaria parasite or as antioxidant

**AUTHOR#1.** Agreed. We anticipated compounds that will be very active but this is what we get at the end of our research.

**REVIEWER#2.** It is hardly to call the functionalized dipeptide scaffold novel unless the authors can demonstrate they have good activity

**AUTHOR#2.** Disagreed. Synthesised compounds can be new or novel without showing impressive biological activity.

**REVIEWER#3.** The screening of a 32-compound library with fixed amino acid backbone (Pro-Ala) is not diverse enough for SAR, and the expansion to a more inclusive compound set seems necessary to discover a more potent inhibitor.

**AUTHORS#3.** I think screening of 32-compound library is fairly OK for biological screening and SAR discussion for an article. However, further expansion will appear in our future articles.

**REVIEWER#4.** It is not entirely clear why the amino acid should be limited to Pro and Ala, at least some other amino acids should be considered.

**AUTHOR#4.** Works are on to expand the project to other amino acids.

**REVIEWER#5.**  The toxicity of these test compounds should be evaluated.
**AUTHOR#5.** Not necessary because the compounds did not exhibit significant activity compare to reference. However important lessons can be learnt from their SAR study.

**REVIEWER#6.**  The discussion in the Synthesis and characterization of new dipeptide derivatives (last paragraph under scheme 2) seems not delivering important information. It is focused on NMR data of the compounds. For example “Significantly, the methyl protons in toluenesulfonyl moiety integrated as singlet for three protons at about 2.40 ppm in the 1H NMR of compounds 9a-p and 10a-p”, why this information is significant? Do the authors try to confirm that the compounds are diastereomerically pure?

**AUTHOR#6.** The statement is modified by doing away with the word ‘significantly’.

**REVIEWER#7.** Compounds 10a-p don’t even have a toluenesulfonyl moiety!

**AUTHOR#7.**  You are right that compounds 10a-p do not have toluenesulfonyl moiety and the mistake is corrected.

**REVIEWER#8.** The authors also tried to convey that a OMe is more deshielded than a Me on the aromatic ring, this is not necessary.

**AUTHOR#8.**  OK. Statement deleted.

**REVIEWER#9.** In addition, the drawings in Scheme 2 need further improvement. First, as a medicinal chemistry manuscript, the identity of the test compounds is very important. No stereochemistry of the amino acid is labelled. The authors should clearly specify the configuration of the compounds since both enantiomers of the amino acids are available and different stereoisomers can alter the activity significantly.

**AUTHORS#9.**  The use of HOBT, a chiral compound as one of the reagents in the reaction supresses isomerisation and hence the generation of a single enantiomer as a major product.

**REVIEWER#10.** In structure 4/5, the X is missing.
**AUTHOR#10.**  Corrected.

**REVIEWER#11.** For the final products, the label should be changed as “9: X= Me, 10: X=NO2”. The current label is very confusing, since R’’ = 9: x = Me is not correct.

**AUTHOR#11.** Corrected.

**REVIEWER#12.** Lipinski’s rule of five is an empirical guideline to assess the drug-likeness of a compound. However, there are many exceptions on both ways. Just using this guideline to evaluate the compounds concludes their drug likeness is not sufficient. One obvious liability is the instability of these peptide drugs in general: they are susceptible to enzymatic degradation. The authors should have a microsomal stability test on them.

**AUTHOR#12.** Since the compounds have low activity, we think it is unnecessary to continue further work on it such as testing for microsomal stability as the reviewer suggested.

**EDITORIAL** **REQUEST**

We have included the relevant information and adjusted financial disclosure statement as you requested. Thank you.
